# Automatic calibration of wellbore pressure wave velocity using perforation-induced shock waves

**DOI:** 10.1038/s41598-026-45490-5

**Published:** 2026-04-16

**Authors:** Xingming Wang, Yuanyuan Yang, Le He, Qiaozhu Wang, Bin He

**Affiliations:** 1 State Key Laboratory of Oil and Gas Reservoir Geology and Exploitation, Chengdu University of Technology, Chengdu, 610059 China; 2 College of Energy, Chengdu University of Technology, Chengdu, 610059 China; 3 TianfuYongxing Laboratory, Chengdu, 610213 China; 4 CNPC Chuanqing Drilling Engineering Company Limited, Chengdu, 610051 China; 5 School of Mechanical and Electrical Engineering, Chengdu University of Technology, Chengdu, 610059 China

**Keywords:** Perforation-induced shock waves, Wellbore pressure wave, Wave velocity calibration, Automatic extraction, Convolution model, Water hammer model, Engineering, Physics, Solid Earth sciences

## Abstract

Conventional wellbore wave velocity estimation mainly relies on water hammer oscillation signals. These signals have a limited frequency range and long oscillation periods. Under complex wellbore conditions, they are easily affected by noise and changes in boundary conditions, which limits the accuracy and stability of wave velocity characterization. To address this limitation, this study focuses on staged hydraulic fracturing in horizontal wells and proposes an automatic method for calibrating wellbore pressure wave velocity using impact pressure waves generated during perforation operations. The impact pressure waves produced during perforation have high frequencies, short pulse durations, concentrated energy, and clear propagation paths. These characteristics provide a signal source with strong physical advantages for detailed wellbore wave velocity characterization. Based on these properties, this study introduces a peak to peak detection approach. This approach shifts wellbore pressure wave velocity calibration from a dependence on overall oscillation features to a physical description based on transient echo time scales. A complete automatic calibration workflow is therefore established. Numerical simulation results show that, for synthetic pressure waves generated by a convolution model, the extracted time intervals between adjacent waves agree well with theoretical values. For individual perforation clusters, the mean absolute error ranges from 1.6 to 1.8 ms, and the root mean square error does not exceed 2.4 ms, indicating millisecond level time extraction accuracy. In addition, simulations based on a water hammer model show that the relative errors of pressure wave peak amplitudes are all below 0.2%. This result confirms the high accuracy of the proposed method from both the time and amplitude perspectives. Field applications indicate that the average wave velocities calculated for individual perforation clusters are mainly concentrated between 1530 and 1555 m/s. The coefficients of variation within clusters are generally below 5%, and the differences in average wave velocity between different stages are less than ± 15 m/s. These results demonstrate good overall consistency and stability. Further statistical analysis shows that the wave velocity distribution at the perforation cluster scale exhibits stable multimodal characteristics. This behavior reflects the possible existence of multiple propagation paths for perforation induced shock waves within the wellbore. The results demonstrate that the proposed method can achieve stable identification of perforation reflected waves and reliable wave velocity calibration under complex wellbore conditions. It provides an effective approach for the quantitative analysis of wellbore acoustic characteristics.

## Introduction

During staged operations in horizontal wells, perforation serves as a critical process that connects the reservoir to the wellbore. Within a very short time, it releases high energy fluids^1–4^ and generates strong wellbore pressure waves. This impact process produces high amplitude transient fluctuations in the wellbore fluid system. As a result, a series of pressure waves propagate along the wellbore and undergo multiple reflections^5^. These transient pressure signals contain diverse physical information, including fluid properties, wellbore parameters, and the coupling state of perforation energy^6^. They serve as important acoustic indicators of perforation energy transfer efficiency, wellbore integrity^7^, and fracture initiation response.

During staged hydraulic fracturing in horizontal wells, the complex wellbore structure and variable fluid boundary conditions, together with high temperature, high pressure, and strong noise during perforation, cause wellbore pressure wave signals to attenuate and distort easily during propagation. Accurate calibration of wave velocity is not only a basic parameter for interpreting wellbore transient signals, but also an important input for subsequent fracture depth inversion and real time fracturing monitoring. According to water hammer wave propagation theory, wave velocity is jointly controlled by fluid compressibility and pipe wall elasticity, and it reflects the combined mechanical behavior of the wellbore and formation system. However, in practical fracturing operations, the wellbore environment is complex and variable. Parameters such as formation elastic modulus, fluid properties, and wellbore stress state are difficult to determine accurately. As a result, wave velocities calculated from theoretical models often differ markedly from field measurements. Because water hammer waves propagate at high velocities, even small errors in wave velocity can lead to noticeable depth shifts, which affect subsequent fracture location and inversion accuracy. Therefore, waves generated by events such as perforation or packer setting^8^ are commonly used in the field to calibrate wave velocity. This practice corrects theoretical calculation errors and improves the reliability of inversion results.

With continuous advances in efficient oil and gas development and well completion technologies, the propagation characteristics of perforation induced shock waves and wave velocity calibration have become active research topics. Deng et al.^9^ investigated the propagation of impact loads in the wellbore and completion system after perforation in ultra deep wells through numerical simulation. Zhang et al.^10^ analyzed the mechanisms of generation, propagation, and attenuation of perforation induced shock waves based on underwater explosion theory. Li et al^11^. proposed a model for pressure wave propagation and attenuation in two phase fluids within the wellbore. Lin et al.^12^ examined the calculation of pressure wave velocity in gas liquid pipelines, which provides reference for similar wave propagation in wellbores. Li et al.^13^ reported that guided waves identifiable in perforation signals with known excitation times can be used to correct velocity models in the wellbore. Ye et al.^14^ inverted radial shear wave velocity profiles around the wellbore using acoustic logging, indicating an alternative direction for velocity calibration. Zhang et al.^15^ found that perforation induced shock waves attenuate at the perforation tunnels and also exhibit changes in wave velocity during axial propagation along the wellbore. Liu et al.^16^ showed that explosive pressure waves in perforating fluids influence wellbore structure and wave velocity propagation characteristics. Zhang et al.^17^ carried out experimental and theoretical analyses on pipelines containing slightly gas bearing liquids. They showed that gas volume fraction and dispersion state in gas liquid two phase flow have a significant influence on pressure wave velocity. As gas content increases or bubble distribution becomes nonuniform, wave velocity decreases markedly or deviates from expected values. A modified wave velocity calculation formula was proposed. Hu et al.^18^ further studied wave velocity from the perspective of water hammer waveform inversion. They established a water hammer pressure wave experimental system and compared four wave velocity calculation methods, including pressure jump point picking, wavelet decomposition with cross correlation, water hammer period analysis, and spectral analysis. These studies provide a theoretical basis for understanding the generation and early stage propagation of wellbore pressure waves and offer valuable reference for wave velocity calibration theory. In addition, previous studies have explored wellbore pressure waves from the perspectives of experimental downlink systems and data-driven recognition, indicating that wellbore pressure waves are not only important carriers of downhole dynamic responses but also exhibit identifiable and predictable temporal features under noisy conditions^19,20^. On the other hand, in the field of geophysical monitoring, the perforation process is widely regarded as a repeatable downhole transient excitation source, and distributed acoustic sensing (DAS) is used to observe and physically interpret the wavefield generated by perforation. Based on DAS observations and numerical modeling, Lellouch et al.^21^ revealed the propagation characteristics of perforation-induced guided waves and their sensitivity to formation structure. Li and Jin^22^ further used the P-waves generated by perforation and their dispersion information to characterize unconventional reservoirs and to extract velocity-related propagation parameters. In terms of source mechanism analysis and inversion frameworks, Bader et al.^23,24^ performed moment tensor inversion of perforation sources based on DAS data, and incorporated perforation-induced signals into full-waveform inversion for reservoir parameter estimation and comparative analysis before and after stimulation. Meanwhile, Zhang et al.^25^ used perforation sources for reservoir characterization and analyzed anisotropy, attenuation, and the sources of uncertainty. These studies demonstrate, from the perspectives of wavefield physics and inversion, that perforation-induced signals can provide important field constraints for medium-parameter interpretation and velocity calibration.

It should also be noted that during engineering processes such as drilling acceleration and reservoir stimulation, the wellbore and formation system is often affected by the coupling of multiple physical processes, including impact loading, confining pressure variation, fracture propagation, and fluid flow in fractures. Yang et al.^26^ investigated the coupling mechanism of impact loading and confining pressure release, and revealed the intrinsic relationship between the improvement of rock breaking efficiency and the variation of stress response under hot dry rock conditions. Tahir and Guo^27^ discussed the effects of hard core defects on fracture propagation patterns and propagation efficiency in CO₂ fracturing of natural hydrogen reservoirs. Li and Liao^28^ analyzed at the microscopic scale the flow resistance mechanisms of displacement fluids in fractures and their effects on pressure gradients and flow behavior. Such coupled effects can alter the transient pressure response of the wellbore and increase the uncertainty of field interpretation. Therefore, it is of practical significance to develop an automatic wave velocity calibration method applicable to field data for completion and stimulation monitoring.

Considering the high frequency, transient, and multiple reflection characteristics of perforation induced shock waves, this study proposes an automatic method for calibrating wellbore pressure wave velocity based on perforation impacts. The method focuses on high amplitude transient pressure waves generated during perforation. Through systematic analysis of shock wave propagation characteristics, it achieves automatic identification of key wellbore reflection information and extraction of time scales, and it quantitatively calibrates wave velocity by incorporating wellbore structural parameters. To verify the reliability and applicability of the method, theoretical analysis and field perforation data are used to systematically evaluate the accuracy and consistency of the proposed wave velocity calculation. Furthermore, statistical analysis is applied to systematically investigate wave velocity distribution characteristics at the perforation cluster scale. This analysis aims to reveal possible multipath propagation behavior of perforation induced shock waves within the wellbore.

## Method

### Propagation process of perforation-induced shock waves

Perforation is a key operational process in which a perforating gun is installed downhole and high energy explosives are detonated inside the casing, creating conductive channels through the steel casing, cement sheath, and surrounding formation. After the perforating charge detonates, chemical energy is rapidly converted into high temperature and high pressure fluids, generating strong shock waves and high velocity metal jets. Because of the pronounced differences in acoustic impedance among the involved media, shock waves are reflected at material interfaces, producing a series of wave components such as the primary wave and reflected waves^29^. Their propagation behavior can be described by the acoustic wave equation:

1 $$\nabla^{2} p = \frac{1}{{c^{2} }}\frac{{\partial^{2} p}}{{\partial t^{2} }}$$

Here, $p$ denotes the disturbance pressure, and $c$ represents the propagation velocity in the medium. This equation describes the propagation of pressure disturbances in time and space, indicating that pressure disturbances travel through the medium in the form of waves, with their velocity jointly determined by the elastic modulus and density of the medium.

**Fig. 1:**
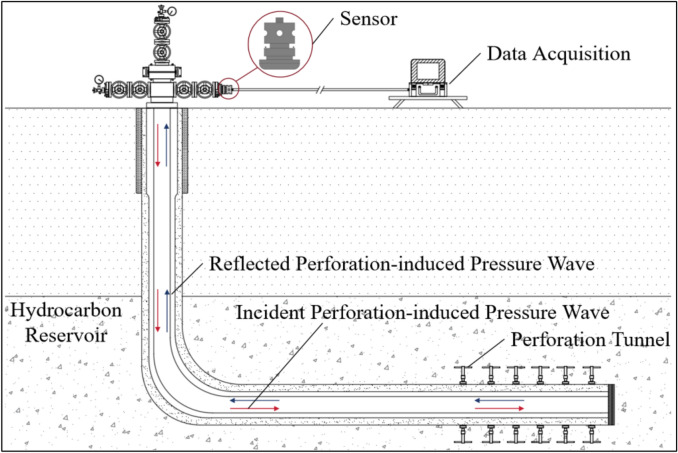
Schematic illustration of the propagation process of perforation-induced shock waves.

Within the wellbore, the shock wave first interacts with the casing and the wellbore fluid, after which part of its energy is transmitted into the cement sheath and the surrounding formation. Owing to differences in physical properties among the layered media, the waveforms attenuate and distort during propagation, and part of the energy is reflected back into the wellbore and recorded by sensors. By analyzing these reflected wave signals, the propagation characteristics and wave velocity of perforation pressure waves can be obtained. The propagation process of the shock wave is illustrated in Fig. 1, and the signals analyzed in this study correspond to the transient response of wellbore pressure during perforation.

### Overall framework for automatic identification

**Fig. 2:**
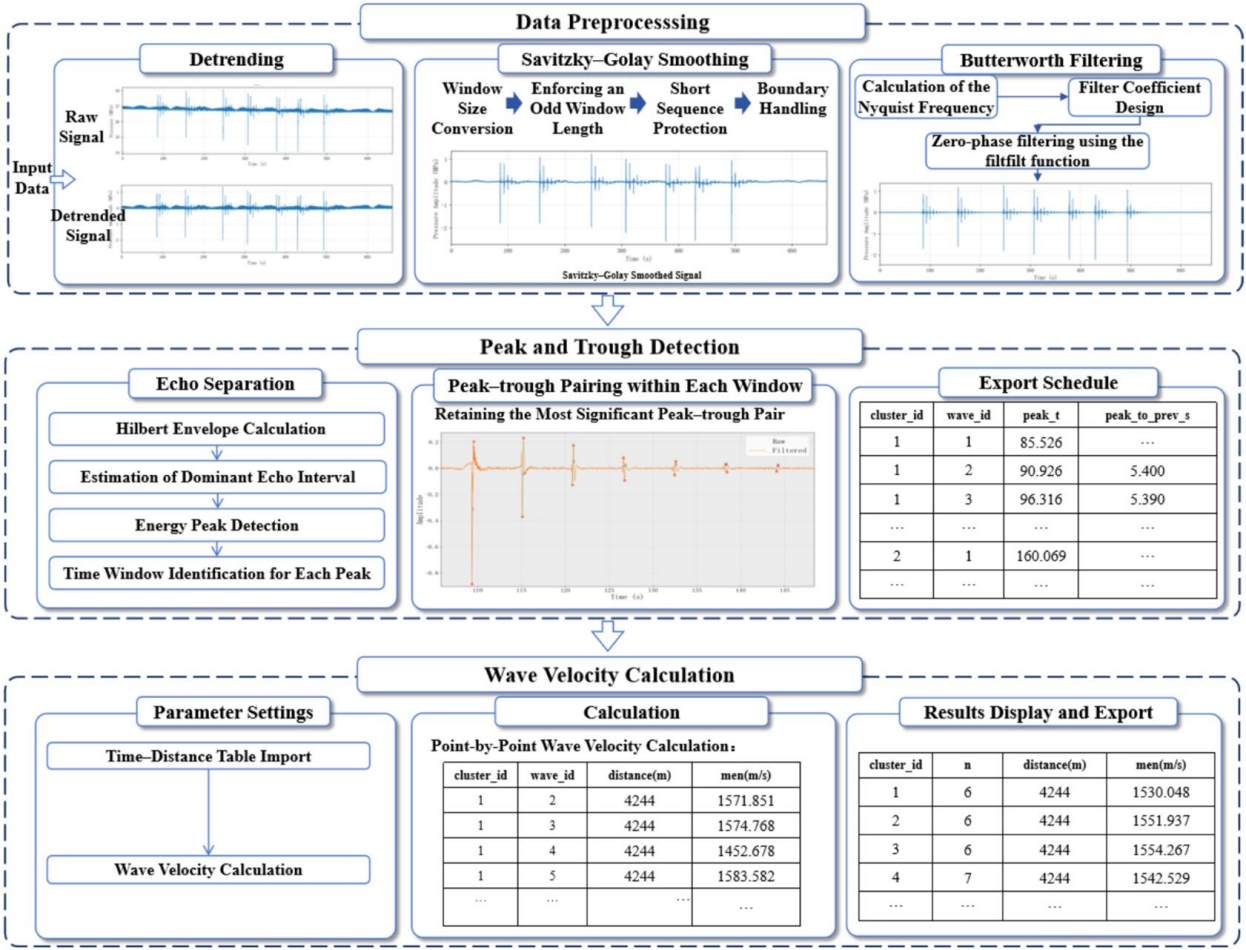
Workflow for automatic calibration of wellbore pressure wave velocity based on perforation-induced shock waves.

To achieve automatic calibration of wellbore pressure wave velocity under perforation impact conditions, this study designs a workflow for identifying wellbore pressure waves and calibrating wave velocity based on perforation-induced shock waves. The method takes high frequency pressure signals as the primary analysis target. By applying systematic filtering and feature extraction to the raw pressure data, it enables automatic identification of the main shock wave and multiple reflected waves, and it completes quantitative wave velocity calibration by incorporating wellbore geometric parameters. The overall workflow is illustrated in Fig. 2 and consists of three core modules: data preprocessing, peak and trough identification, and wave velocity calibration.

In the data preprocessing stage, three successive steps are applied according to the characteristics of the data: detrending, Savitzky–Golay smoothing, and Butterworth filtering. Detrending is used to remove baseline drift components from the signal, allowing the pressure curve to return to a stable reference level. Savitzky–Golay smoothing suppresses random noise through local polynomial regression. In nonstationary signal processing, it preserves the local shape and extreme positions of transient shock waves, thereby avoiding distortion of the main shock wave peak^22^. Butterworth filtering further extracts effective energy components within the target frequency band and removes interference from instruments and the surrounding environment. After preprocessing, the waveform features of the main shock wave and its multiple reflections become clearer, providing high quality input for subsequent identification.

In the peak and trough identification stage, the Hilbert envelope $e(t) = |H\{ y(t)\} |$ is calculated from the preprocessed signal $y(t)$, and $e(t)$ is then smoothed. Envelope peak detection is then performed on $e(t)$, and the peak prominence threshold is defined adaptively based on a robust noise scale: $pro\min ence \ge \max (1.8*MAD(e),0.02*\max (e))$. A minimum spacing is imposed between adjacent envelope peaks to avoid repeated segmentation. For each envelope peak, the left and right boundaries of the window are determined at the positions where the envelope decays to $\alpha$ times the peak value, and the window is further extended at both ends to cover the complete echo wave packet, yielding each echo window $[t_{s} ,t_{e} ]$. For each echo window, the global maximum and global minimum within the window are taken as the candidate peak and trough, respectively, and the extrema are searched again within a local neighborhood around the candidate points for refinement, so as to reduce the extremum shift caused by discrete sampling and noise. The validity of each extremum pair is determined jointly by temporal and amplitude constraints. The temporal constraint is defined using a half-period window: $\Delta t \in [HALF\_MIN\_MS,HALF\_MAX\_MS]$. The amplitude constraint is adaptively defined based on a robust noise scale: $\sigma_{n} = MAD(y_{w} )$ is calculated for the signal $y_{w}$ within the window, and the peak-to-trough amplitude difference $A = |y_{T} - y_{p} |$ is required to satisfy $A \ge \max (AMP\_MIN,c*\sigma_{n} )$.With this adaptive threshold setting that varies with the noise level, the algorithm can maintain a consistent decision criterion across windows with different noise intensities, thereby improving robustness.

In the wave velocity calculation stage, the time intervals between adjacent waves in each cluster and the corresponding plug distances are extracted from the identified peak and trough sequences, and the perforation wave velocity is then calculated. During calculation, reference perforation numbers and correction parameters are introduced. Wave velocity is calculated individually for each cluster and each wave segment, followed by unified calibration and statistical averaging to reduce the influence of noise and systematic errors. The final wave velocity results reflect the propagation behavior between perforations and provide quantitative support for connectivity analysis of the wellbore and fracture system.

**Fig. 3:**
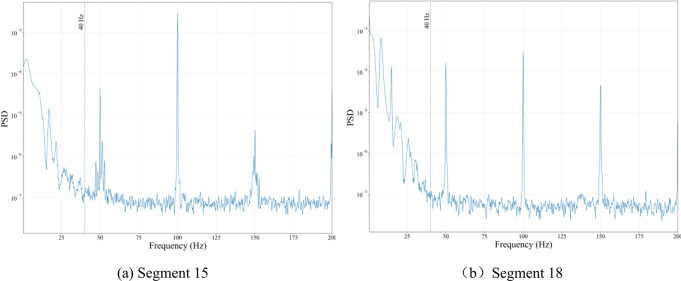
Spectral energy distribution of the raw wellhead pressure signal.

To avoid subjective selection of the filtering parameters, we performed PSD analysis on the raw wellhead pressure signals from representative perforation event windows, and the results are shown in Fig. 3 and Table 1 The spectra of Segment 15 and Segment 18 both show that the main energy is concentrated below 40 Hz, while narrowband interference peaks are also visible near 50 Hz, 100 Hz, and 150 Hz. Therefore, a default band of 0.5 to 40 Hz was adopted in the subsequent processing as the effective frequency band, so that the main signal components could be preserved while high-frequency noise and narrowband interference were suppressed.

**Table 1:** Proportion of spectral energy.

Frequency	Segment 15	Segment 18
≤ 40 Hz	0.520	0.959
40–100 Hz	0.062	0.017
100–200 Hz	0.214	0.014

For reproducibility, all default and used parameters of the automated workflow are summarized in Table 2.

**Table 2:** Key parameters used in the automated workflow.

Module	Parameter	Value used	Unit	Notes
Data input	FS	1000	Hz	Sampling frequency
Detrending	DETREND_MODE	linear	–	Linear detrending
	CENTER_BY	median	–	Median centering for robustness
SG smoothing	SG_WIN_MS	51	ms	SG window; internally converted to samples and forced to be odd with length adaptation
	SG_POLY	3	–	SG polynomial order
SG safeguard	MIN_SEG_PTS_SG	7	points	Skip SG for very short segments
Butterworth	hp_eff	0.5	Hz	Effective cutoff is computed as hp_eff = max(HP_HZ,0.5)
	lp_eff	40	Hz	Effective cutoff is computed as lp_eff = max(LP_HZ,40)
	Filtfilt (method = “gust”)	enabled	–	Zero-phase filtering to avoid phase delay in timing
Half-cycle constrain	HALF_MIN_MS	8	ms	Minimum peak-trough separation
	HALF_MAX_MS	300	ms	Maximum peak-trough separation
Pairing threshold	AMP_MIN	0.03	Mpa	Minimum amplitude threshold for peak–trough pairing
Noise metric	_mad()	MAD		Noise estimated by MAD, used in adaptive thresholding
Onset detection	ENV_SMOOTH_MS	25	ms	Hilbert-envelope smoothing window
	ON_Z	5	–	Onset threshold multiplier
	DWELL_MS	15	ms	Required dwell time above threshold
First-wave correction	FIRST_WIN_MS	200	ms	Search window for the first-wave pair
	FIRST_TOP_FRAC	0.45	–	Strength ratio threshold for the dominant first extreme
	FIRST_LATENCY_MS	25	ms	Minimum latency after onset
	GUARD_BEFORE_S	0.2	s	Guard time after onset
Extreme refinement	REFINE_MS	30	ms	± refinement window around candidate extrema
Echo segmentation	ECHO_MIN_SEP_MS	900	ms	Minimum separation between envelope peaks
	ECHO_MIN_WIDTH_MS	40	ms	Minimum echo-window width
	ECHO_ALPHA	0.15	–	Alpha-span ratio for defining window boundaries
	ECHO_PROM_Z	3.8	–	Envelope-peak prominence threshold

#### Principle of detrending

The essence of detrending is to remove low frequency and slowly varying components from the original signal that are not relevant to the target analysis, so that the remaining signal more clearly represents short period or transient features. In wellbore pressure signals, these low frequency trends usually originate from changes in pumping pressure, variations in tubing friction, temperature effects, or system drift, and they tend to mask high frequency fluctuations such as perforation impacts.

The principle of detrending can be understood by assuming that the observed signal can be expressed as:

2 $$x\left( t \right) = s\left( t \right) + d\left( t \right)$$

Here, $s\left( t \right)$ denotes the high frequency component, and $d\left( t \right)$ represents the slowly varying trend term, which can be approximated as $d\left( t \right) \approx at + b$. The parameter $a$ describes the linear drift in the signal, while $b$ represents the baseline offset. By estimating $d\left( t \right)$ and removing it from the original signal, the following expression can be obtained:

3 $$x_{{{\mathrm{d}}etrend}} \left( t \right) = x\left( t \right) - d\left( t \right)$$

As a result, the signal becomes more stable and its mean approaches zero, which facilitates subsequent processing steps such as filtering, envelope extraction, peak and trough detection, and arrival time estimation.

#### Principle of Savitzky–Golay smoothing

Savitzky–Golay smoothing is a filtering method based on local polynomial fitting. Its basic idea is to fit a low order polynomial within a sliding window using the least squares method and to use the fitted value at the central point as the smoothed output. Compared with simple moving average filtering, which only performs weighted averaging of the signal, the Savitzky–Golay method uses local polynomial approximation to suppress random noise while preserving signal details such as peaks, troughs, and transient variations to the greatest extent.

For a data point located at time $t_{0}$, a sliding window with a length of 2*k* + 1 is selected:

4 $$\left\{ {y\left( {t_{0} - k} \right), \ldots ,y\left( {t_{0} } \right), \ldots ,y\left( {t_{0} + k} \right)} \right\}$$

It is assumed that the signal within this window can be approximated by an *m*-order polynomial:

5 $$y\left( t \right) \approx a_{0} + a_{1} \left( {t - t_{0} } \right) + a_{2} (t - t_{0} )^{2} + \cdots + a_{m} (t - t_{0} )^{m}$$

The coefficient vector $${\mathbf{a}} = \left[ {\begin{array}{*{20}c} {a_{0} ,a_{1} , \ldots ,a_{m} } \\ \end{array} } \right]^{T}$$ is obtained using the least squares method:

6 $${\mathbf{a}} = ({\mathbf{X}}^{T} {\mathbf{X}})^{ - 1} {\mathbf{X}}^{T} {\mathbf{y}}$$

Here:

7 $${\mathbf{X}} = \left[ {\begin{array}{*{20}c} 1 & { - k} & {( - k)^{2} } & \cdots & {( - k)^{m} } \\ 1 & { - \left( {k - 1} \right)} & { - (k - 1)^{2} } & \cdots & { - (k - 1)^{m} } \\ \vdots & \vdots & \vdots & \ddots & \vdots \\ 1 & k & {k^{2} } & \cdots & {k^{m} } \\ \end{array} } \right]$$

The smoothed value is given by the value of the fitted polynomial at the central point, that is $t$ = $t_{0}$.

8 $$\hat{y}\left( {t_{0} } \right) = a_{0}$$

#### Principle of Butterworth filtering

The pressure signals recorded during perforation contain multiple noise components, including detonation noise, mechanical vibration, and environmental interference. These factors introduce high frequency fluctuations and low frequency drift, which reduce the accuracy of subsequent peak detection and wave velocity calculation. To extract the main energy characteristics of shock wave propagation, filtering is applied to the raw signal to suppress noise and retain effective information within the target frequency range.

The basic principle of filtering is to use the frequency selective property of linear systems, so that specific frequency components of the input signal are preserved or attenuated.According to their frequency response characteristics, commonly used filters include low pass, high pass, band pass, and band stop filters. In this study, the energy of perforation pressure signals is mainly concentrated in the low and middle frequency range, whereas the high frequency components are dominated by random noise. Therefore, a Butterworth filter is selected for signal preprocessing^23,24,30,31^.

The Butterworth filter is characterized by a maximally flat amplitude response. It exhibits no ripples in the passband and a smooth, monotonic transition band, which allows effective suppression of high frequency noise while maintaining signal smoothness. Its amplitude frequency response can be expressed as:

9 $$|H\left( {j\omega } \right)|^{2} = \frac{1}{{1 + \left( {\frac{\omega }{{\omega_{c} }}} \right)^{2n} }}$$

Here, $H\left( {j\omega } \right)$ denotes the frequency response function of the filter, $\omega_{c}$ is the cutoff angular frequency, and $n$ is the filter order. When $\omega \ll \omega_{{\mathrm{c}}}$,$\left| {{\mathrm{H}}\left( {{\mathrm{j}}\omega } \right)} \right| \approx 1$, indicating that the signal experiences almost no attenuation within the passband; when $\omega \gg \omega_{{\mathrm{c}}}$,$\left| {{\mathrm{H}}\left( {{\mathrm{j}}\omega } \right)} \right| \approx 0$, and high frequency noise is effectively suppressed. As the filter order ${\mathrm{n}}$ increases, the transition near the cutoff frequency becomes steeper, while the phase delay increases accordingly.

In practical data processing, the Butterworth filter is typically implemented in digital form, where the discretized transfer function is derived from its analog form using bilinear transformation.:

10 $$H\left( z \right) = \frac{{b_{0} + b_{1} z^{ - 1} + \cdots + b_{n} z^{ - n} }}{{1 + a_{1} z^{ - 1} + \cdots + a_{n} z^{ - n} }}$$

Here, $b_{i}$ and $a_{i}$ are filter coefficients, which are determined by the cutoff frequency and the filter order.

#### Principle of wellbore wave velocity calculation

The propagation velocity of pressure waves in the wellbore is jointly influenced by fluid compressibility, fluid density, and the coupling characteristics between the wellbore and the formation. When pressure waves propagate within the wellbore, their energy is mainly transmitted through the fluid in the form of longitudinal waves. At the same time, elastic responses are induced in the casing, the wellbore wall, and the surrounding formation, which reduces the propagation velocity relative to the acoustic velocity in an ideal fluid. For an incompressible fluid, when deformation of the wellbore wall is neglected, the pressure wave propagation velocity can be approximated in terms of the fluid bulk modulus $K$ and density $\rho$ as:

11 $$v = \sqrt {\frac{K}{\rho }}$$

Here, $K$ denotes the fluid bulk modulus (Pa), and $\rho$ denotes the fluid density (kg/m^3^). This expression describes the intrinsic acoustic velocity of pressure disturbances in a fluid medium and represents the ability of the fluid to respond to internal compression and expansion.

However, under actual perforation conditions, the wellbore system does not behave as an ideal rigid boundary. Under the action of pressure waves, the wellbore wall, casing, and surrounding formation undergo small elastic deformations. As a result, part of the wave energy is consumed by medium deformation rather than propagation, which leads to a reduction in the effective wave velocity. To account for this coupling effect, the corrected wellbore wave velocity can be expressed as:

12 $$\frac{1}{{v^{2} }} = \frac{1}{{a^{2} }} + \frac{D}{{E_{t} }}$$

Here, $a = \sqrt {\frac{K}{\rho }}$ denotes the intrinsic acoustic velocity of the fluid, and $E_{t}$ represents the equivalent elastic modulus of the formation (Pa), which characterizes the ability of the formation to resist deformation. The parameter $D$ is the wellbore-formation coupling coefficient, and its value depends on factors such as wellbore diameter, casing thickness, formation Poisson’s ratio, and porosity.

This expression indicates that lower formation stiffness and stronger wellbore-formation coupling lead to a lower pressure wave propagation velocity. This relationship reflects the influence of wellbore-formation interaction on wave propagation characteristics. In practical applications, the formation elastic modulus can be estimated through acoustic measurements or numerical simulations, which allows further refinement of the wave velocity model and improves calculation accuracy.

On the other hand, transient pressure disturbances generated by perforation impacts propagate along the wellbore toward the wellhead. During propagation, reflections occur at the well bottom, at packers, and at locations where the wellbore geometry changes, which leads to a superposition of multiple reflected waves in the wellhead pressure records. After perforation, the pressure sequence measured at the wellhead exhibits a series of successive waveform peaks and troughs, with a stable time interval between adjacent waves. This time interval reflects differences in the propagation paths of pressure waves within the wellbore and can be used to characterize the actual propagation velocity in the medium.If the propagation distance $L$ from the perforation interval to the reflecting interface is known, the actual wave velocity in the wellbore can be calculated by measuring the time difference ${\Delta }t$ between adjacent waves:

13 $$v = \frac{2L}{{{\Delta }t}}$$

This expression is derived from the time–distance method based on round-trip wave propagation, in which the wave travels a round-trip path length of *2L* within the wellbore. Moreover, this formulation does not depend on the specific wellbore geometry or on waveform details. Instead, it relies solely on the path difference and arrival time difference between adjacent waves, which provides high stability and practical applicability in field operations. When the reflecting interface is known, the wellbore pressure wave velocity can be robustly calibrated by measuring the arrival time difference between the direct wave and the reflected wave. This provides reliable fundamental parameters for subsequent depth inversion and hydraulic fracturing monitoring.

### System architecture and module functions

**Fig. 4:**
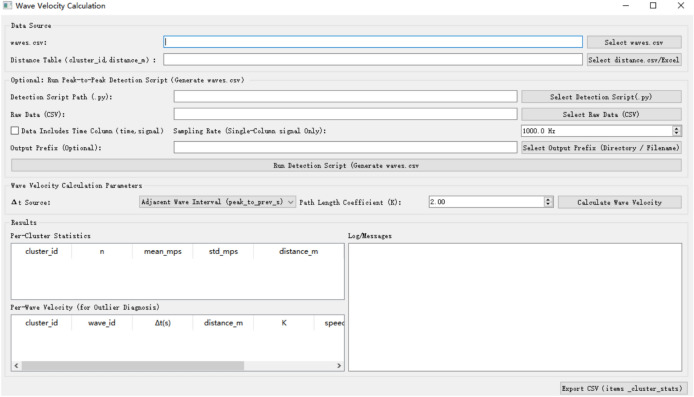
Wave velocity calculation interface.

To enable automated implementation of the peak-to-peak detection method and visualization of results, a perforation-induced shock wave velocity calculation system was developed in a Python environment. The system adopts a Python and PyQt technical framework and integrates signal processing algorithms with graphical interface design, thereby realizing a complete workflow from data input to wave velocity calculation. A three-layer modular architecture is employed, consisting of a data support layer, a functional logic layer, and a user interaction layer. Standardized data interfaces are used to enable data transfer and function calls between layers, forming a closed-loop processing pipeline from raw signal input and feature identification to result output. The wave velocity calculation interface is shown in Fig. 4.

1. User interaction layer

The user interaction layer is implemented using a PyQt graphical interface and serves as the primary interface between the system and the user. This layer provides visual functions such as data loading, parameter configuration, waveform inspection, and result export. Users can inspect preprocessing effects in real time, select target perforation intervals, and perform peak-to-peak detection within the interface. After detection, the system automatically generates wave velocity calculation results and graphical displays, enabling full visualization and interactive control from signal input to result output.

(2) Functional logic layer

The functional logic layer is the core component of the system and integrates three main modules: signal preprocessing, peak-to-peak detection, and wave velocity calculation. The preprocessing module incorporates multiple signal enhancement functions to suppress low-frequency drift and random noise, thereby improving the signal-to-noise ratio. The peak-to-peak detection module serves as the key algorithm unit. Through the GUI, it supports interactive window selection, real-time annotation, and automatic result storage, and it performs peak and trough identification, wave cluster indexing, and result output within user-defined perforation intervals.

(3) Data support layer

The data support layer provides the foundation for system operation and is responsible for raw signal import, data standardization, and result storage. The system supports data input in multiple formats, automatically identifies time-series or single-channel signal structures, and determines the sampling frequency based on the input type or user-defined settings. This layer also manages structured result storage, including peak-to-peak detection outputs, wave velocity statistics tables, and annotated image files, providing data support for subsequent analysis and visualization.

### Principle of method validation

#### Convolution model

To verify the reliability of the proposed wellbore pressure wave velocity calibration procedure, a convolution model was constructed based on the linear time-invariant characteristics of the wellbore-fluid system. During propagation of transient pressure disturbances generated by perforation, the system response can be regarded as the convolution superposition of a source wavelet and a sequence of reflections from multiple wellbore interfaces. Because the wellbore structure remains stable over short time intervals, the fluid can be treated as a weakly compressible medium, and pressure wave propagation satisfies the principle of superposition, the convolution framework can accurately describe the propagation and reflection behavior of perforation-induced shock waves.

**Fig. 5:**
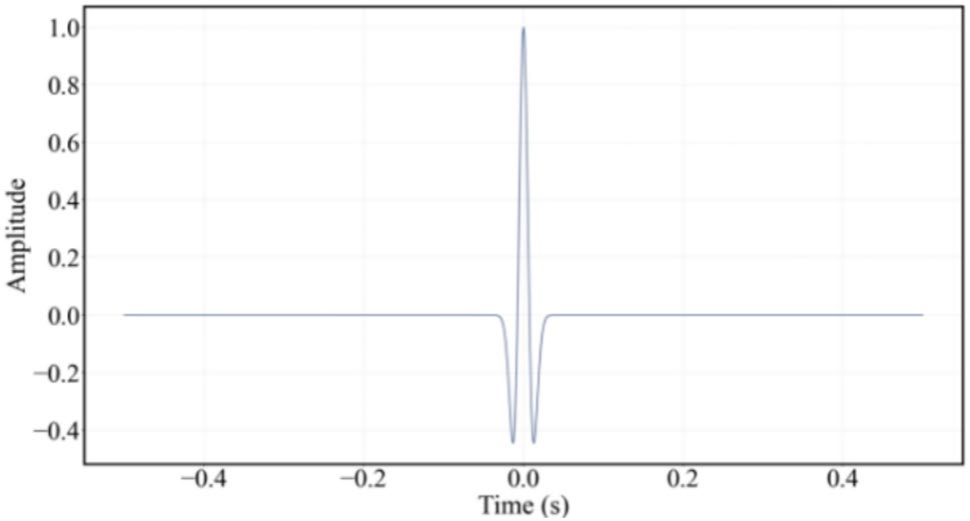
Ricker wavelet.

**Fig. 6:**
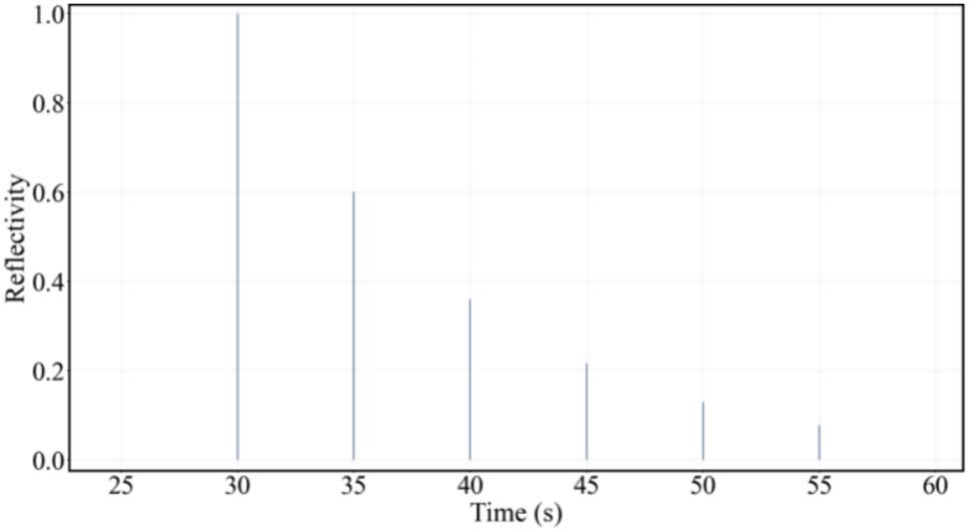
Configuration of reflecting interfaces.

The input signal $s\left( t \right)$ of the model uses a Ricker wavelet to approximate the initial impact generated by perforation, and its waveform is shown in Fig. 5. This wavelet exhibits zero phase, a single dominant peak, and a concentrated frequency spectrum, which closely matches the shape of the main pressure peak observed in actual perforation signals. Perforation clusters, the well bottom, and other structural interfaces within the wellbore cause partial energy reflections, and their reflection strength is described by the reflection coefficient $r_{i}$. By representing all reflecting interfaces as an impulse sequence, as shown in Fig. 6, the reflection response function of the system can be obtained as:

14 $$r\left( t \right) = \mathop \sum \limits_{i = 1}^{N} r_{i} \delta \left( {t - \tau_{i} } \right)$$

Here, $\tau_{i} = \frac{{2L_{i} }}{v}$ denotes the round-trip travel time associated with the $i$-th reflecting interface, $L_{i}$ represents the interface position, and $v$ is the prescribed wellbore wave velocity.

15 $$p\left( t \right) = s\left( t \right)*r\left( t \right) = \mathop \sum \limits_{i = 1}^{N} r_{i} s\left( {t - \tau_{i} } \right)$$

The convolution model can generate simulated pressure waveforms with multiple echo clusters, multiple attenuated echoes, and clearly separated intervals, which effectively capture the multiple reflection characteristics that occur in the wellbore after perforation impacts. By adjusting the reflection coefficients, interface positions, and noise levels, data sets representing different scenarios can be constructed for subsequent validation of the automatic wave velocity calibration algorithm.

#### Water hammer model

To evaluate the accuracy and robustness of the proposed automatic calibration method for perforation-induced shock wave velocity, a one-dimensional wellbore water hammer propagation model was constructed. Under known wave velocity, wellbore structure, and boundary conditions, perforation pressure wave signals were synthesized and used as input data for the automatic calibration procedure. By comparing the prescribed wave velocity in the model with the velocity automatically inverted by the algorithm, the performance of the method under ideal water hammer conditions was evaluated, providing a theoretical basis for subsequent field applications.

The fluid inside the wellbore is treated as a compressible single-phase liquid, and the influence of pipe wall elasticity on wave propagation velocity is taken into account. According to water hammer theory, the one-dimensional transient governing equations can be expressed as:

16 $$\frac{\partial H}{{\partial t}} + \frac{{a^{2} }}{gA}\frac{\partial Q}{{\partial x}} + RQ\left| Q \right| = 0$$

17 $$\frac{\partial Q}{{\partial t}} + gA\frac{\partial H}{{\partial x}} = 0$$

Here, $H$ denotes the hydraulic head, $Q$ is the volumetric flow rate, $A$ is the cross-sectional area, and $a$ is the one-dimensional equivalent wave velocity. The parameter $R = f/\left( {2gDA^{2} } \right)$, where $D$ is the internal pipe diameter and $f$ is the friction factor. The term $RQ\left| Q \right|$ represents the distributed friction term, which describes the energy dissipation of pressure waves during propagation.

Under the system of Eqs. (16 and 17), the two characteristic line directions of one-dimensional water hammer propagation are given by:

18 $$\frac{dx}{{dt}} = \pm a$$

**Fig. 7:**
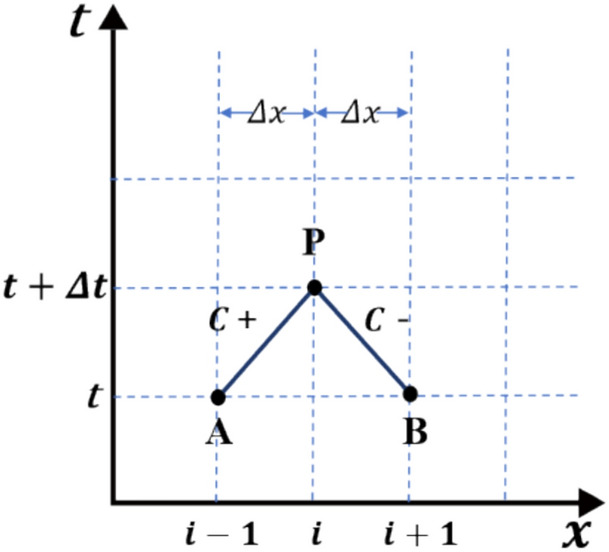
Schematic diagram of characteristic lines.

The spatial step is set to $dx$, and the time step is set to $dt = dx/a$, so that the characteristic lines (as shown in Fig. 7) fall exactly on adjacent grid points and an explicit scheme without numerical dispersion is obtained. For an interior node (i,j), we have:

19 $$C_{P} = H_{i - 1}^{j - 1} + BQ_{i - 1}^{j - 1} - RQ_{i - 1}^{j - 1} \left| {Q_{i - 1}^{j - 1} } \right|$$

20 $$C_{M} = H_{i + 1}^{j - 1} - BQ_{i + 1}^{j - 1} + RQ_{i + 1}^{j - 1} \left| {Q_{i + 1}^{j - 1} } \right|$$

21 $$Q_{i}^{j} = \frac{{C_{P} - C_{M} }}{2B}$$

22 $$H_{i}^{j} = \frac{{C_{P} + C_{M} }}{2}$$

Here, $$B = c/\left( {gA} \right)$$.

## Results and validation

### Method validation

#### Validation using the convolution model

Based on the established convolution model, controllable synthetic data were constructed to evaluate the reliability of the proposed wellbore pressure wave velocity calibration method under different operating conditions and to verify its effectiveness. By specifying reflecting interface locations, propagation distances, and time delays within the convolution model, artificial pressure signals containing multiple reflections were generated and fed into the developed wave velocity calibration workflow. The deviation between the intra-cluster wave interval extracted by the algorithm and the prescribed theoretical value was then analyzed to assess the accuracy and stability of the method.

**Fig. 8:**
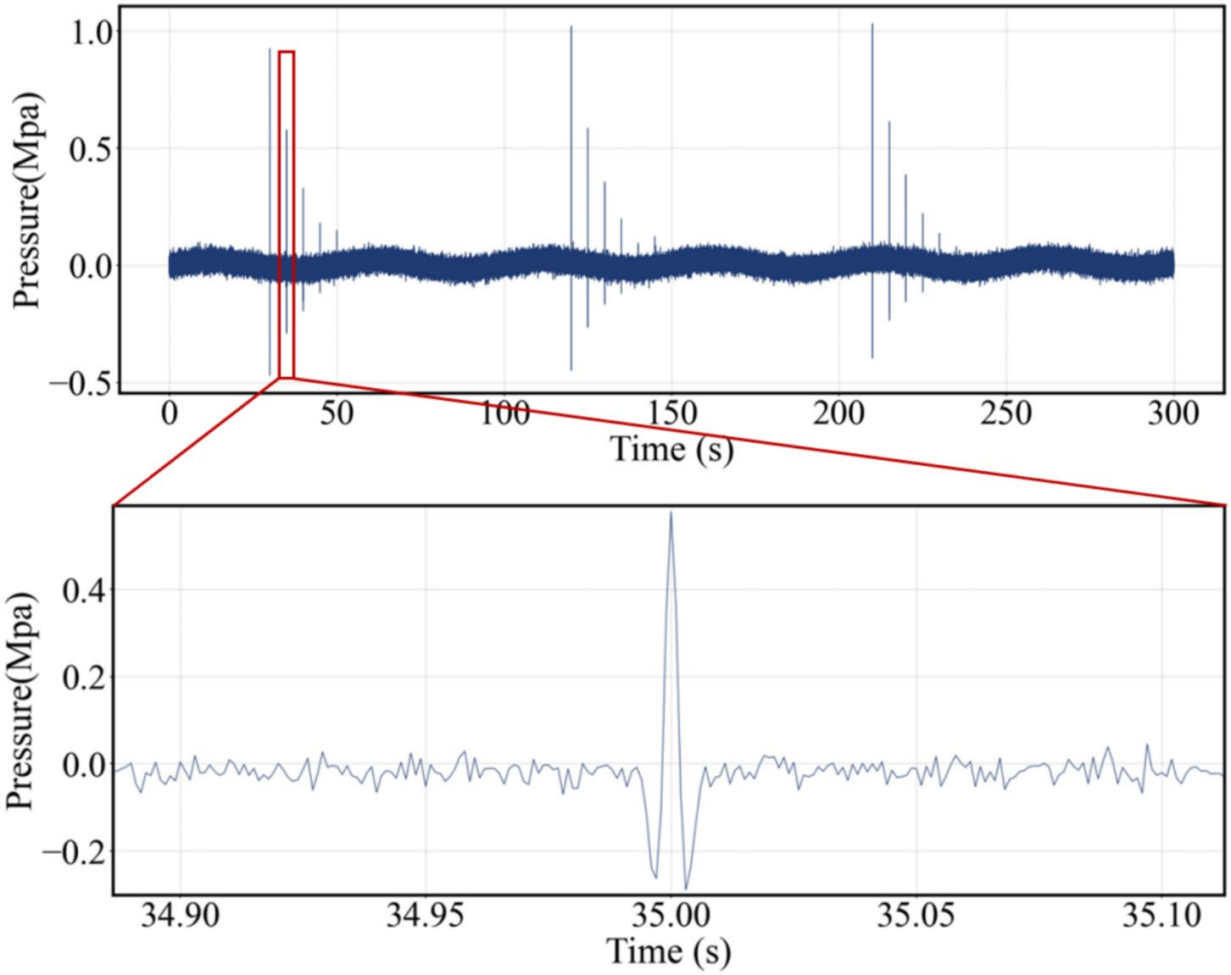
Simulated echo pressure waveforms.

During model construction, multiple reflecting interfaces were defined according to the typical spacing between perforation clusters, and different reflection strengths were assigned to each interface. By varying the number of interfaces, their distances, and the amplitude attenuation patterns, wellbore reflection scenarios with different levels of structural complexity were generated. These interface parameters were used to generate impulse sequences with different delay positions, and the convolution model automatically superimposed source wavelets with corresponding amplitudes according to these delays, producing multi-echo pressure waveforms with clear temporal structures, as shown in Fig. 8. To improve the consistency between the simulated data and field pressure records, Gaussian white noise and low-frequency baseline drift of specified intensity were added to the generated waveforms to mimic instrument noise and long-term acquisition trends at the wellhead. As a result, the simulated waveforms exhibit both ideal structural features and realistic engineering disturbances.

**Fig. 9:**
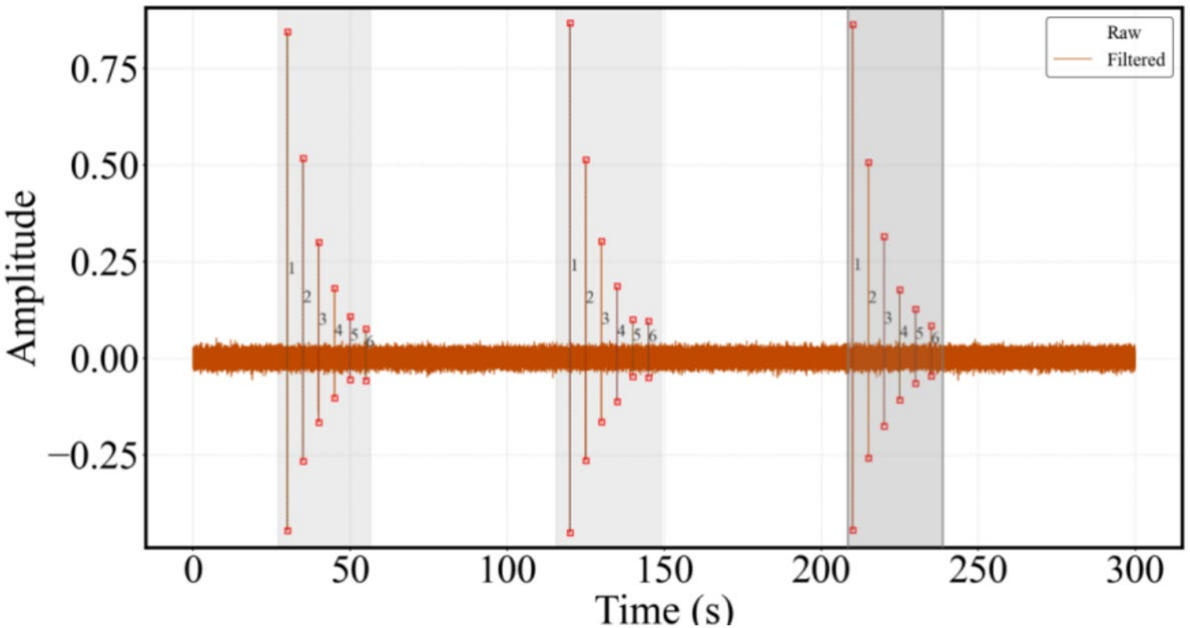
Identification results.

The generated simulated waveforms are directly used as input data for validation within the proposed wave velocity calibration workflow. The entire validation procedure follows the same processing steps as those applied to field data, including signal preprocessing, extremum detection, waveform clustering, echo grouping, and propagation time extraction. Because the true propagation time associated with each reflecting interface in the simulated data is 5 s, the wave interval times extracted by the algorithm can be directly compared with the prescribed theoretical delays to quantify error sources and evaluate the temporal resolution capability of the algorithm. The identification results are shown in Fig. 9.

To quantitatively illustrate the performance of echo identification, the peak times, trough times, and the corresponding time intervals $\Delta t$ of each echo cluster generated by the convolution model were statistically analyzed and summarized in Table 3. This table clearly reveals the arrival-time patterns of successive echoes and provides a solid numerical basis for subsequent wave velocity calculations.

**Table 3:** Identified time intervals between successive echoes.

cluster_id	wave_id	peak_to_prev_s(s)
1	1	
1	2	5
1	3	5.003
1	4	4.996
1	5	5.000
1	6	4.999
2	1	
2	2	5
2	3	5
2	4	4.997
2	5	5.002
2	6	4.996
3	1	
3	2	5.003
3	3	4.997
3	4	5
3	5	5
3	6	5.003

**Fig. 10:**
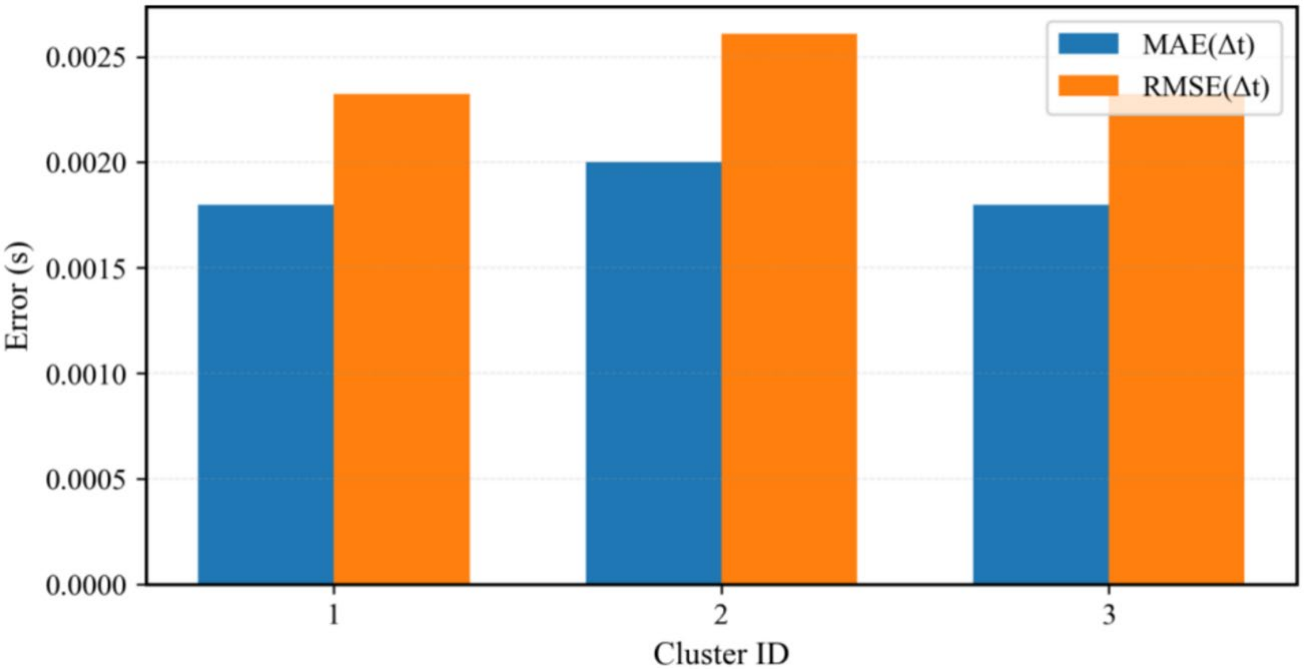
Bar charts of MAE and RMSE for each cluster.

To further evaluate the overall performance of the proposed method, the mean absolute error (MAE) and root mean square error (RMSE) were employed to quantitatively assess the identification errors of adjacent-wave time intervals under different simulated scenarios. Figure 10 illustrates the MAE and RMSE of inter-wave intervals for each cluster. The results show that the MAE values of all three clusters are controlled at approximately $2.0 \times 10^{ - 3}$ s, while the RMSE remains within about $2.4 \times 10^{ - 3} \sim 2.7 \times 10^{ - 3}$ s. The overall error level is relatively low, indicating that the algorithm demonstrates good consistency and stability across different clusters.

**Fig. 11:**
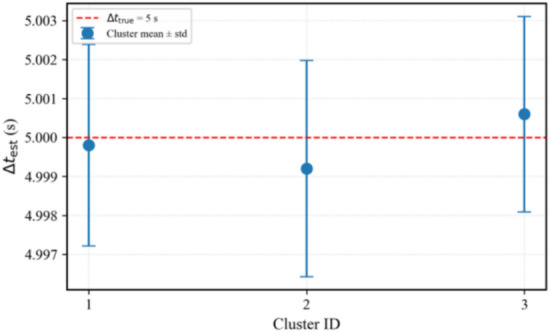
Mean values with ± 1 standard deviation error bars.

To evaluate the concentration of the identification results, Fig. 11 presents the mean values and standard deviations of the inter-wave time intervals for each cluster. The mean values of all three clusters closely match the true preset value of 5 s, with deviations on the order of approximately $10^{ - 3}$ s. The standard deviations are consistently distributed around $2.0 \times 10^{ - 3}$ s, indicating good stability and repeatability of multiple-echo identification within each cluster. These results demonstrate that the proposed workflow can reliably extract the temporal structure of adjacent echoes within clusters and remains robust under varying reflection energies and amplitude attenuation conditions.

**Fig. 12:**
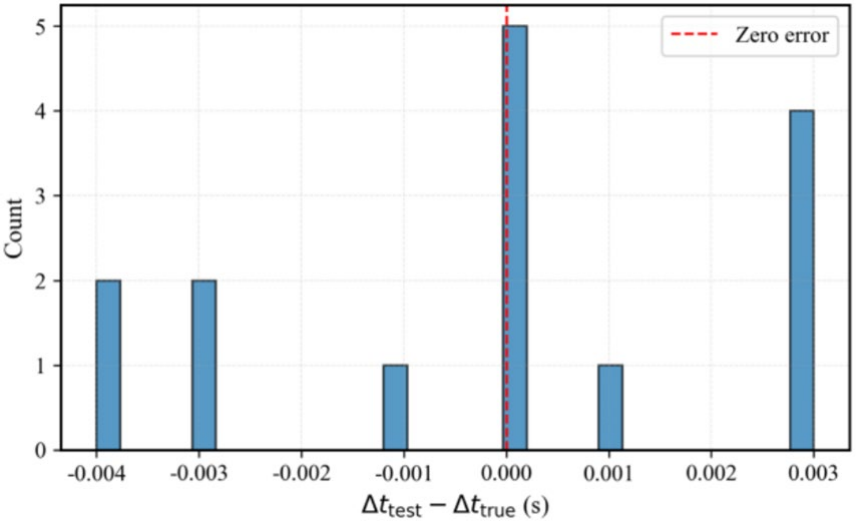
Histogram of identification errors.

The error histogram shown in Fig. 12 further illustrates the distribution of the extracted time interval ${\Delta }t_{{{\mathrm{t}}est}} - {\Delta }t_{{{\mathrm{t}}rue}}$. Most errors are concentrated around zero, with only a few samples exhibiting a slight deviation of approximately $\pm 3.0 \times 10^{ - 3}$ s, and no systematic bias is observed. This indicates that the entire identification workflow does not introduce systematic time drift due to peak-through detection or pairing errors. The residual errors are mainly attributed to minor sampling noise or interpolation uncertainty.

Meanwhile, this study conducted a noise-level sensitivity test on convolution-based synthetic pressure signals. Under the condition that the waveform ground truth remained unchanged, the noise level was set to three cases, namely 10 dB, 5 dB, and 0 dB, and an automated workflow was used to extract the intervals between adjacent echoes. Table 4 presents the statistics of this test. It can be seen that the echo intervals can be stably extracted under all three noise conditions, and the MAE of the interval time remains at the millisecond level.

**Table 4:** Summary statistics of the sensitivity test.

SNR_dB	MAE	STD
10	0.0079	0.010
5	0.0062	0.009
0	0.0065	0.009

The results obtained from the synthetic data demonstrate that, under ideal and well controlled conditions with known true propagation times, the proposed wave velocity calibration workflow achieves millisecond level accuracy in time extraction. The results from all three clusters converge steadily to the theoretical values, and no evident misidentification or systematic bias is observed. These results confirm the reliability and robustness of the method in identifying inter echo time intervals. They also provide a solid basis for the subsequent application of the method to field perforation induced pressure signals.

#### Validation using a water hammer model

**Fig. 13:**
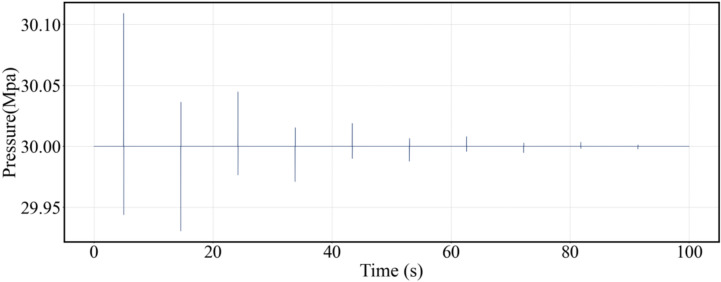
1-D water-hammer wellhead pressure response used as a baseline reference (not the primary validation for perforation transients).

In the model, the wellbore length $L = 6000\,m$ and the wave velocity $c = 1250m/s$ are specified, with the damping parameter set to $dx = 2m$, and the excitation applied at the central time $t_{0} = 0.2s$ of the Gaussian pulse. The simulated wellhead pressure curve is shown in Fig. 13.

Under the present simulation condition, the wellbore length $L$ and the wave speed $c$ are known parameters, so the theoretical round-trip travel time $T = 2L/c$ can be calculated directly. Therefore, the key point of the validation is to determine whether the automatically identified peak time intervals agree with the theoretical values. When the identified results are stable and show close agreement with the theoretical values, this demonstrates the correctness of the reflection identification and wave-speed calibration procedure. In this case, further wave-speed inversion would lead to the same result and is therefore not repeated.

The theoretical round-trip travel time at the wellhead is given by:

23 $$T = \frac{2 \times 6000}{{1250}} = 9.6{\mathrm{s}}$$

**Fig. 14:**
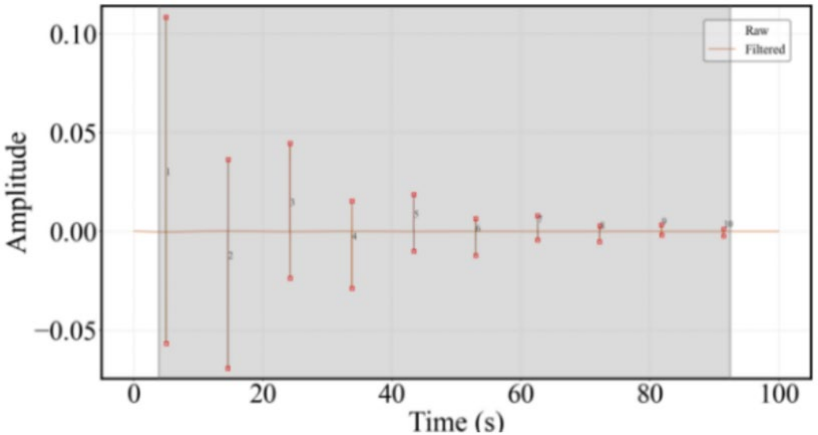
Identification results of pressure waves generated by the water-hammer model.

Therefore, under ideal conditions, the peak time intervals between successive reflections should approach 9.6 s. Figure 14 shows the identification results of the simulated pressure waves generated by the water-hammer model within the wave-speed calibration workflow.

**Fig. 15:**
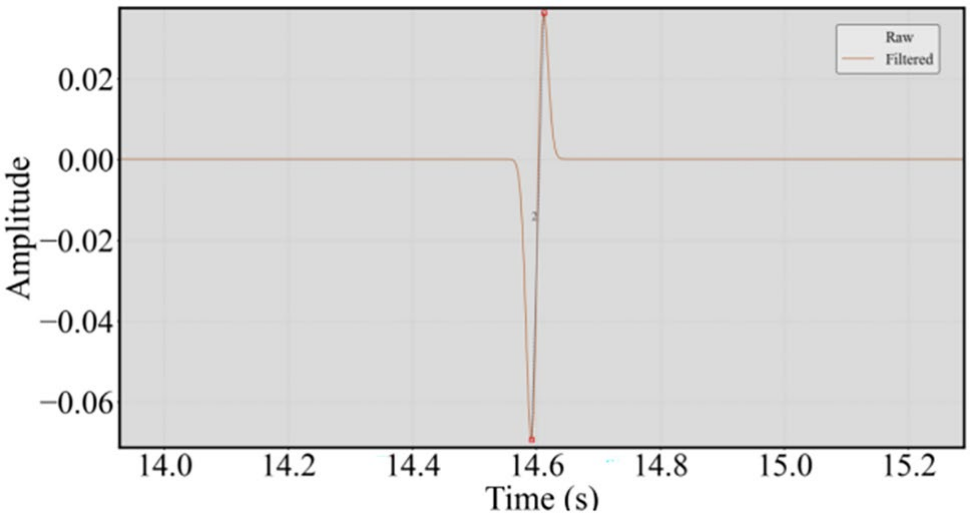
Enlarged view of the identification result for the second echo.

Figure 15 presents a local magnified view of the second echo in the filtered transient pressure signal. The algorithm can accurately locate the peak and trough of this echo, which are directly indicated by annotations in the figure. This result demonstrates that the proposed identification method can effectively capture the main features of the echo, providing a reliable basis for subsequent time-difference calculation and wave-speed calibration.

Table 5 lists the statistical timing results of the identified echoes, including peak times, trough times, and peak-to-peak intervals between adjacent echoes. It can be observed that the echo arrival times exhibit a clear increasing trend, which is highly consistent with the round-trip propagation behavior of water-hammer waves in the wellbore. This table provides clear and quantifiable time inputs for wave-speed calibration and serves as an important basis for subsequent inversion calculations.

**Table 5:** Identification results.

cluster_id	wave_id	peak_t(s)	trough_t(s)	peak_to_prev_s(s)
1	1	4.992	5.012	–
1	2	14.592	14.612	9.600
1	3	24.192	24.212	9.600
1	4	33.792	33.812	9.600
1	5	43.392	43.412	9.600
1	6	52.992	53.012	9.599
1	7	62.592	62.612	9.600
1	8	72.192	72.212	9.599
1	9	81.792	81.812	9.600
1	10	91.392	91.412	9.599

The results show that the multiple reflection periods obtained from numerical simulations are fully consistent with the theoretical predictions, with relative errors between successive peaks smaller than 0.2%. Therefore, the proposed wave velocity calibration procedure exhibits high accuracy and stability for perforation-induced transient signals and can serve as a reliable method for wellbore wave velocity calibration.

### Validation based on field data

#### Data preprocessing

**Fig. 16:**
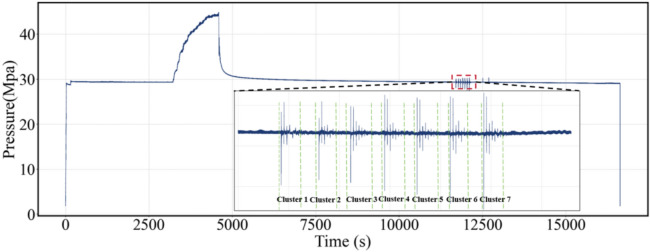
Raw pressure signal of the perforation cluster.

This study uses pressure signals measured during a perforation operation in an oil and gas well as the research object, with a sampling rate of 1000 Hz. The signals capture the high-amplitude shock wave generated at the instant of perforation and its multiple reflections within the wellbore. Owing to the complex field operating conditions, the raw signals are inevitably affected by fluid disturbances and random noise, which results in slow baseline drift and high-frequency spikes. Figure 16 shows the raw perforation pressure signal acquired at the field site.

**Fig. 17:**
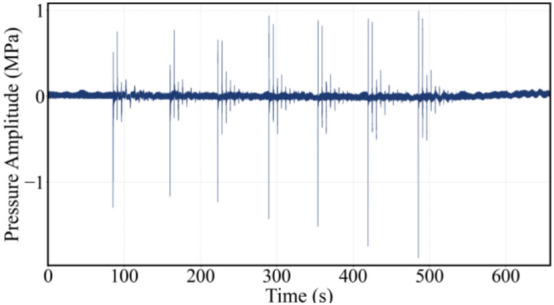
Result after detrending.

**Fig. 18:**
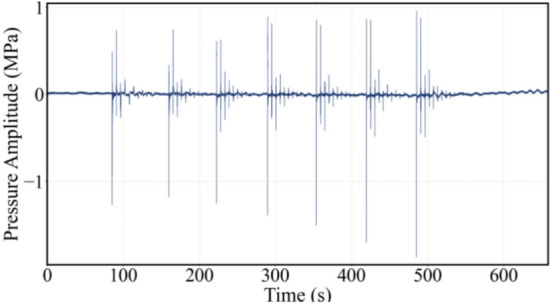
Result after Savitzky Golay smoothing.

**Fig. 19:**
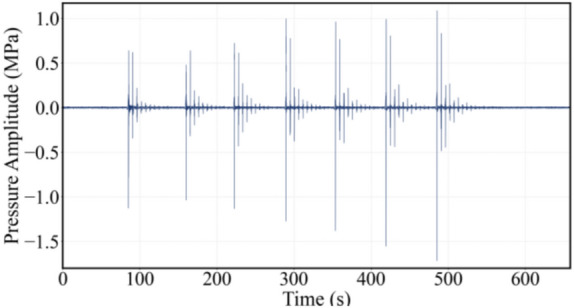
Result after Butterworth filtering.

To improve signal quality and highlight the main reflected features of the perforation shock wave, the signal is first processed using linear detrending to remove low-frequency drift, so that the waveform fluctuates around a near-zero mean level.After this processing, the signal baseline becomes stable, as shown in Fig. 17. Next, Savitzky–Golay smoothing is applied to suppress random high-frequency noise, while reducing spikes and preserving the shape and timing characteristics of the main shock wave peak, as shown in Fig. 18. Finally, a Butterworth filter is applied to extract the main energy components of the signal. The cutoff frequency is set to 40 Hz, and a forward and backward filtering scheme with zero phase shift is used to eliminate phase distortion, as shown in Fig. 19.

**Fig. 20:**
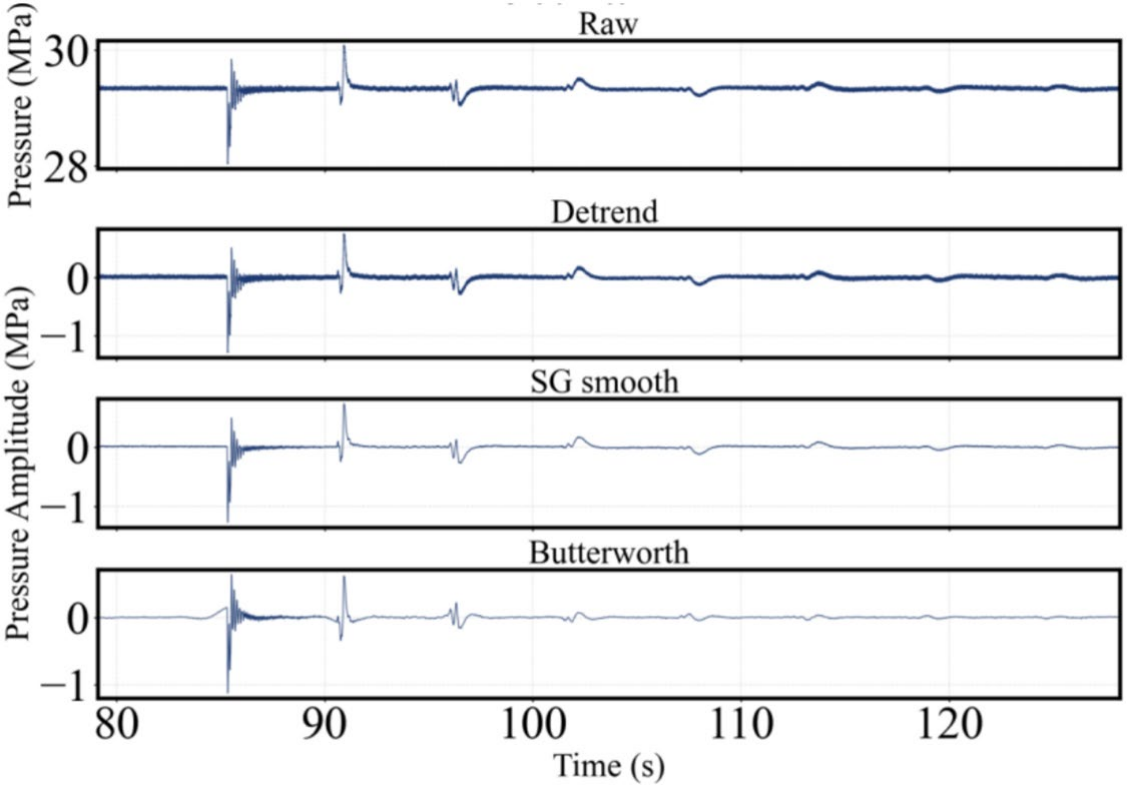
Enlarged processed result of perforation cluster 1 in segment 18.

After the three processing steps, the shock wave and its multiple reflection features become clearly visible in the signal. The background noise is greatly reduced, and the signal to noise ratio is significantly improved. Figure 20 shows a local enlarged view of perforation cluster 1. The main shock wave shape is clearly identified, and the energy of the subsequent reflected waves shows evident attenuation, which provides a reliable basis for subsequent peak to peak detection and wave speed calculation.

**Fig. 21:**
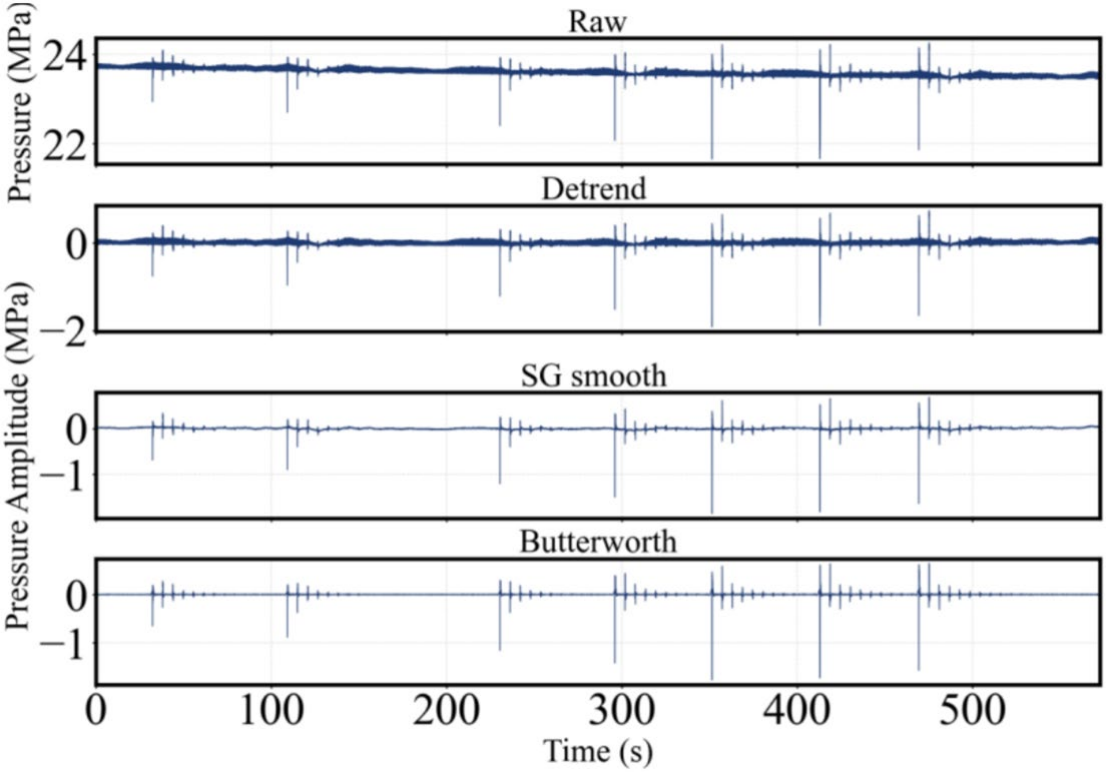
Data from segment 15.

To further verify the applicability of the preprocessing method in different perforation segments, Fig. 21 presents the processing results of the perforation data from segment 15 under the same procedures. It can be observed that although the energy amplitude and attenuation behavior differ among perforation segments, the preprocessing procedure consistently reveals the main shock wave and its multiple reflections, which provides a reliable basis for subsequent consistency analysis.

#### Results of the identification algorithm

To verify the applicability and identification accuracy of the peak and valley detection algorithm for field perforation data, this study implements the algorithm in an engineering form on the Python platform. The program uses high frequency perforation pressure signals as input and applies a graphical interface for band selection and result display. During practical operation, the user loads the preprocessed signal through the interface and selects the target perforation time window to trigger automatic detection. After the detection process is completed, the program automatically generates result files and annotated images. The result files record the peak time, valley time, and peak to peak interval for each wave in each cluster, which facilitates subsequent statistical analysis and wave speed evaluation.

**Fig. 22:**
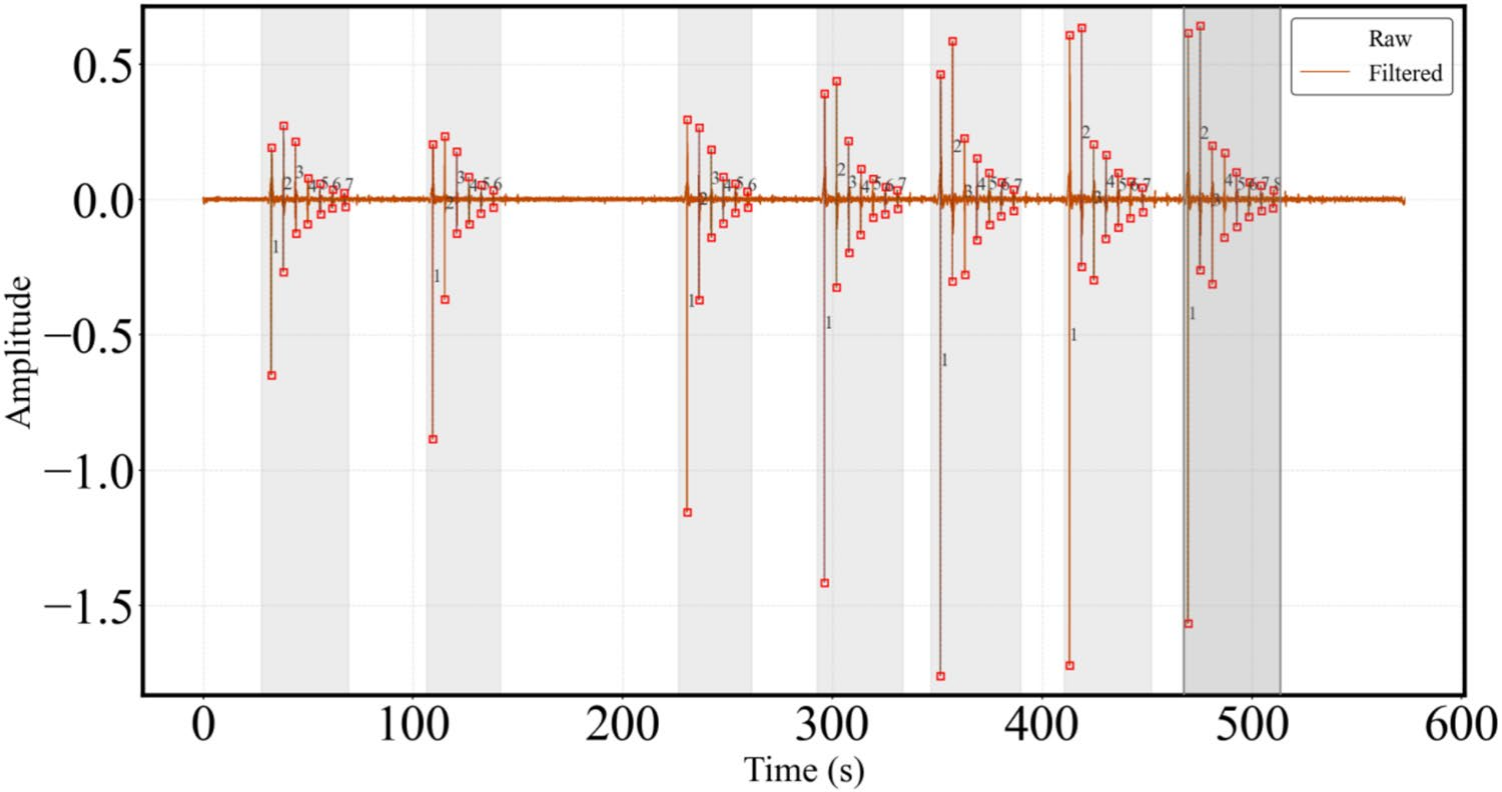
Identification results for segment 15.

**Fig. 23:**
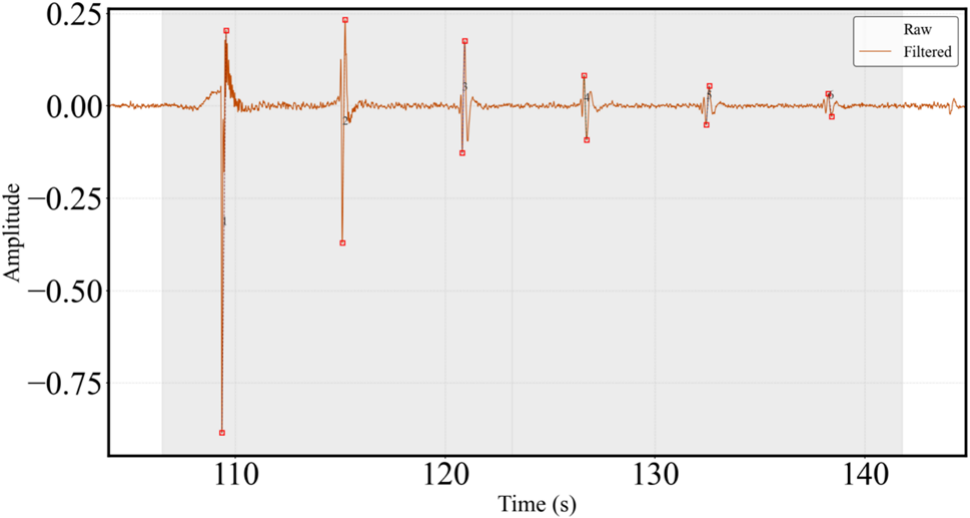
Peak and valley identification results for perforation cluster 2 in segment 15.

Figure 22 shows the identification results for the perforation cluster in segment 15. It can be observed that the main perforation peak is accurately located, and the subsequent reflected waves exhibit a step by step attenuation trend. The peak and valley markers are clear, as shown in Fig. 23, and the reflection sequence structure is complete. This indicates that the algorithm can still stably extract effective features in well segments where the signal energy is weak and attenuation is rapid.

**Fig. 24:**
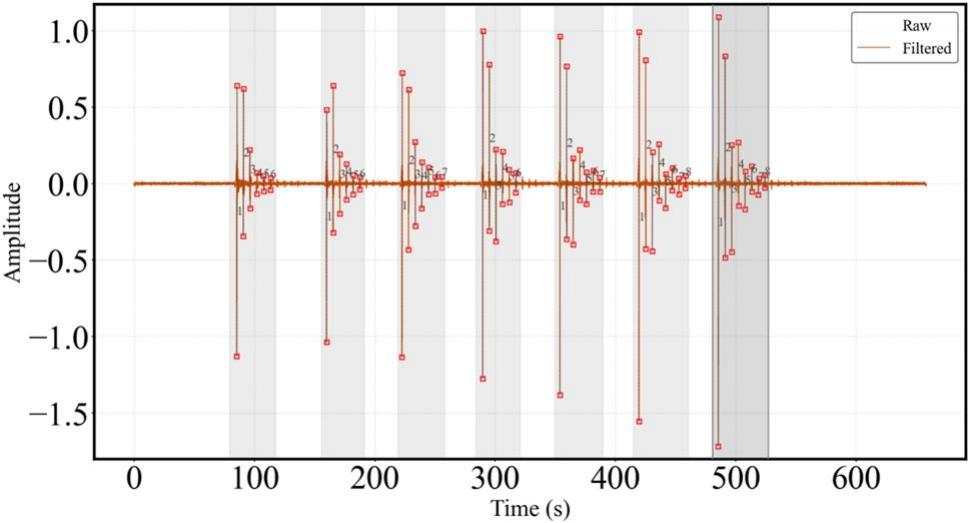
Identification results of segment 18.

Figure 24 presents the identification results of the perforation data from segment 18. Compared with segment 15, the reflected waves of the perforation clusters in segment 18 show higher energy, and the periodic structure is more evident. The algorithm can also accurately identify the peaks and valleys of successive reflected waves. The time intervals between the reflected waves remain highly consistent, which further confirms the applicability and robustness of the algorithm under different perforation energy conditions.

Tables 6 and 7 list the peak and trough identification results and the interval $\Delta t$ between adjacent main peaks for the perforation data of segment 15 and segment 18, respectively.The results show that both data sets contain multiple clusters and multiple levels of reflected waves, with the values of $\Delta t$ mainly distributed between 5.2 s and 6.3 s, while the variation of $\Delta t$ within each cluster is small and shows a stable periodic pattern. This indicates that the algorithm can reliably extract the time features of the reflection paths and provides a sound basis for wave speed inversion.

**Table 6:** Peak and trough identification results and intervals between adjacent main peaks for each perforation cluster in segment 15.

cluster_id	wave_id	peak_t(s)	trough_t(s)	win_start(s)	win_end(s)	peak_to_prev_s(s)
1	1	32.560	32.398	27.650	74.041	–
1	2	38.284	38.145	27.650	74.041	5.724
1	3	44.000	44.176	27.650	74.041	5.716
1	4	50.074	49.816	27.650	74.041	6.074
1	5	55.676	55.928	27.650	74.041	5.602
1	6	61.834	61.509	27.650	74.041	6.158
1	7	67.323	67.707	27.650	74.041	5.489
1	8	73.586	73.199	27.650	74.041	6.263
2	1	109.554	109.357	105.551	153.141	–
2	2	115.229	115.093	105.551	153.141	5.675
2	3	120.926	120.810	105.551	153.141	5.697
2	4	126.613	126.740	105.551	153.141	5.687
2	5	132.584	132.439	105.551	153.141	5.971
2	6	138.256	138.418	105.551	153.141	5.672
2	7	144.250	144.069	105.551	153.141	5.994
2	8	149.906	150.157	105.551	153.141	5.656
3	1	230.742	230.618	223.666	267.815	–
3	2	236.450	236.338	223.666	267.815	5.708
3	3	242.137	242.275	223.666	267.815	5.687
3	4	247.840	247.949	223.666	267.815	5.703
3	5	253.776	253.646	223.666	267.815	5.936
3	6	259.458	259.610	223.666	267.815	5.682
4	1	296.289	296.168	291.324	334.327	–
4	2	301.986	301.874	291.324	334.327	5.697
4	3	307.674	307.806	291.324	334.327	5.688
4	4	313.649	313.472	291.324	334.327	5.975
4	5	319.299	319.505	291.324	334.327	5.650
4	6	325.346	325.101	291.324	334.327	6.047
4	7	330.959	331.200	291.324	334.327	5.613
5	1	351.602	351.471	344.647	389.371	–
5	2	357.300	357.180	344.647	389.371	5.698
5	3	362.981	363.107	344.647	389.371	5.681
5	4	368.960	368.773	344.647	389.371	5.979
5	5	374.590	374.800	344.647	389.371	5.630
5	6	380.681	380.406	344.647	389.371	6.091
5	7	386.243	386.509	344.647	389.371	5.562
6	1	413.119	412.989	409.439	457.029	–
6	2	418.818	418.702	409.439	457.029	5.699
6	3	424.497	424.625	409.439	457.029	5.679
6	4	430.469	430.288	409.439	457.029	5.972
6	5	436.101	436.323	409.439	457.029	5.632
6	6	442.175	441.910	409.439	457.029	6.074
6	7	447.720	448.030	409.439	457.029	5.545
7	1	469.606	469.474	465.629	518.953	–
7	2	475.312	475.188	465.629	518.953	5.706
7	3	480.978	481.116	465.629	518.953	5.666
7	4	486.952	486.773	465.629	518.953	5.974
7	5	492.576	492.806	465.629	518.953	5.624
7	6	498.695	498.402	465.629	518.953	6.119
7	7	504.210	504.539	465.629	518.953	5.515
7	8	510.421	510.032	465.629	518.953	6.211

**Table 7:** Peak and trough identification results and intervals between adjacent main peaks for each perforation cluster in segment 18.

cluster_id	wave_id	peak_t(s)	trough_t(s)	win_start(s)	win_end(s)	peak_to_prev_s(s)
1	1	85.526	85.350	80.286	125.208	–
1	2	90.926	90.768	80.286	125.208	5.400
1	3	96.316	96.544	80.286	125.208	5.390
1	4	102.159	101.889	80.286	125.208	5.843
1	5	107.519	107.976	80.286	125.208	5.360
1	6	113.682	113.152	80.286	125.208	6.163
1	7	118.921	119.414	80.286	125.208	5.239
2	1	160.069	159.885	156.203	198.369	–
2	2	165.466	165.370	156.203	198.369	5.397
2	3	170.855	171.000	156.203	198.369	5.389
2	4	176.548	176.363	156.203	198.369	5.693
2	5	181.869	182.098	156.203	198.369	5.321
2	6	187.725	187.399	156.203	198.369	5.856
2	7	192.938	193.219	156.203	198.369	5.213
3	1	222.795	222.575	217.476	260.960	–
3	2	228.165	228.020	217.476	260.960	5.370
3	3	233.522	233.682	217.476	260.960	5.357
3	4	239.210	239.039	217.476	260.960	5.688
3	5	244.542	244.740	217.476	260.960	5.332
3	6	250.282	250.039	217.476	260.960	5.740
3	7	255.595	255.816	217.476	260.960	5.313
4	1	289.830	289.600	282.701	332.115	–
4	2	295.188	295.045	282.701	332.115	5.358
4	3	300.542	300.710	282.701	332.115	5.354
4	4	306.242	306.046	282.701	332.115	5.700
4	5	311.554	311.768	282.701	332.115	5.312
4	6	317.315	317.063	282.701	332.115	5.761
4	7	322.594	322.819	282.701	332.115	5.279
4	8	328.408	328.100	282.701	332.115	5.814
5	1	353.958	353.721	349.245	396.023	–
5	2	359.318	359.478	349.245	396.023	5.360
5	3	364.667	364.836	349.245	396.023	5.349
5	4	370.362	370.172	349.245	396.023	5.695
5	5	375.692	375.880	349.245	396.023	5.330
5	6	381.430	381.199	349.245	396.023	5.738
5	7	386.708	386.918	349.245	396.023	5.278
5	8	392.484	392.240	349.245	396.023	5.776
6	1	419.654	419.415	415.129	462.566	–
6	2	424.986	425.188	415.129	462.566	5.332
6	3	430.740	430.525	415.129	462.566	5.754
6	4	436.047	436.299	415.129	462.566	5.307
6	5	441.836	441.570	415.129	462.566	5.789
6	6	447.108	446.801	415.129	462.566	5.272
6	7	452.364	452.630	415.129	462.566	5.256
6	8	458.148	457.922	415.129	462.566	5.784
7	1	485.625	485.365	481.014	533.721	–
7	2	490.953	491.154	481.014	533.721	5.328
7	3	496.715	496.485	481.014	533.721	5.762
7	4	502.007	502.251	481.014	533.721	5.292
7	5	507.853	507.554	481.014	533.721	5.846
7	6	513.071	513.409	481.014	533.721	5.218
7	7	519.007	518.574	481.014	533.721	5.936
7	8	524.163	523.857	481.014	533.721	5.156

#### Wave velocity extraction and validation

Based on the previously identified main peak and main trough for each cluster, the propagation velocity of the perforation induced pressure wave in the wellbore can be further calculated. This study uses the geometric distance data of perforation clusters and applies the developed wave velocity calculation program to obtain the propagation velocity and statistics for each cluster. The program uses the time interval between adjacent echoes as the time difference input and applies the echo method with a path factor of K = 2 to automatically calculate the wave velocity $c_{i} = KL/{\Delta }t_{i}$ for each single sample, and then derives the mean wave velocity, the standard deviation, and the sample count for each perforation cluster.

Table 8 presents the wave velocity calculation results for each perforation cluster in segment 15. Overall, the mean wave velocities of all clusters are concentrated in the range from 1532 to 1553 m/s, and the differences among clusters are small, which indicates that the propagation velocity in this wellbore section is relatively uniform. Among them, Cluster 3 has the highest mean wave velocity and the smallest standard deviation, which indicates that its echo times are the most stable and its propagation paths are the most consistent, making it the most representative cluster for the wave velocity in this section. By comparison, Clusters 1 and 7 show slightly larger standard deviations, which reflects the presence of a small number of wave velocity outliers within these clusters. Even so, their mean values remain within a narrow range of ± 10m/s, and no pronounced deviation is observed. The cluster level wave velocity distribution in Section 15 is stable with limited variation, and all clusters fall within the theoretical wave velocity range, which confirms the reliability of the wave velocity extraction process.

**Table 8:** Statistical results of wave velocity for each perforation cluster in segment 15.

cluster_id	n	mean(m/s)	std(m/s)	distance(m)
1	6	1541.874	69.514	4459.000
2	6	1543.106	40.718	4459.000
3	5	1553.224	28.5894	4459.000
4	6	1544.633	48.279	4459.000
5	6	1546.330	55.382	4459.000
6	6	1548.076	54.829	4459.000
7	7	1532.241	69.733	4459.000

Table 9 summarizes the wave velocity results for each cluster in segment 18. Similar to segment 15, the average wave velocities in segment 18 are mainly concentrated within the range of 1530 to 1555 m/s, with only small variations among clusters. Cluster 1 exhibits the largest standard deviation, indicating relatively larger fluctuations in echo identification within this cluster. Cluster 3 shows the highest average wave velocity and, consistent with segment 15, remains one of the most stable clusters in terms of wave velocity within the segment. Overall, the cluster level results for segment 18 maintain good concentration, with the mean values of all clusters falling close to the theoretical wave velocity range and no obvious abnormal clusters observed.

**Table 9:** Statistical results of wave velocity for each perforation cluster in segment 18.

cluster_id	n	mean(m/s)	std(m/s)	distance(m)
1	6	1530.048	93.575	4244.000
2	6	1551.937	67.285	4244.000
3	6	1554.267	53.353	4244.000
4	7	1542.529	64.395	4244.000
5	7	1544.332	60.493	4244.000
6	7	1546.443	71.284	4244.000
7	7	1546.413	89.620	4244.000

## Discussion

### Statistical consistency and stability analysis

**Fig. 25:**
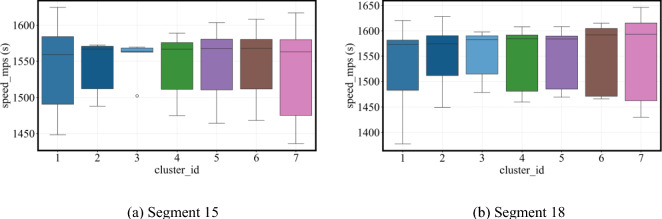
Box plot of wave speed for perforation clusters.

Figure 25 shows the box plots of wave speed for each perforation cluster in Segment 15 and Segment 18. The medians of wave speed in both segments are stably distributed between 1530 and 1555 m/s, and the difference between segments is very small, which indicates good overall consistency of propagation speed among different perforation segments. The box heights in both segments are generally small, and the interquartile ranges are concentrated, which indicates stable identification of echo intervals and low dispersion of wave speed within each cluster. A few clusters show slightly longer whiskers, which reflects locally weaker echo amplitude or the influence of changes in wellbore conditions, but the number of outliers is very small and the wave speed values still fall within the theoretical speed range.

**Fig. 26:**
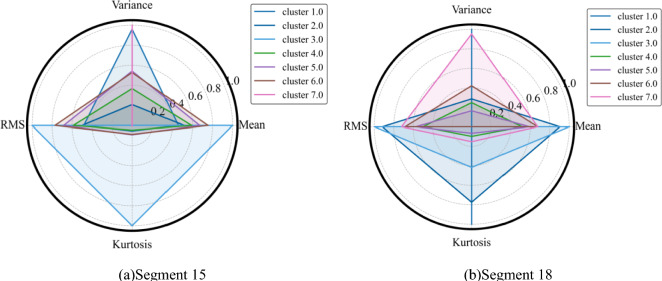
Statistical radar plots for each perforation cluster.

This study uses four statistical indicators, including mean, variance, root mean square error, and kurtosis, to perform a multi dimensional evaluation of wave speed for each cluster, as shown in Fig. 26. The mean represents the central level of wave speed and is a key metric for evaluating consistency between different stages. Variance and root mean square error jointly describe the stability of wave speed and the overall level of fluctuation. Kurtosis is used to identify sharp peak distributions or abnormal values.

Through analysis of statistical radar plots for each perforation cluster in segment 15 and segment 18, it can be observed that cluster level wave speeds in both stages show highly consistent overall patterns across mean, root mean square error, variance, and kurtosis, with only a few clusters showing normal level variation in variance and kurtosis. The overall shapes of the radar plots for the two segments largely overlap, indicating that the statistical characteristics of wave speed remain highly consistent across different perforation segments, which reflects the stability and robustness of the proposed echo identification and wave speed calculation method when applied across segments. Combined with the theoretical range of wave speed described earlier, the cluster level wave speeds extracted from both segments fall within the theoretical limits, which further verifies the accuracy and physical validity of the proposed method.

**Fig. 27:**
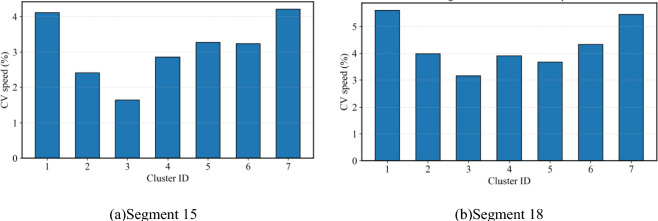
Coefficients of variation of wave speed for each cluster.

Figure 27 shows the coefficients of variation of wave speed for each cluster in segment 15 and segment 18. It can be seen that the coefficients of variation for segment 15 are mainly within the range of 2 to 4%, whereas those for segment 18 are slightly higher overall and mostly fall within the range of 3 to 5%. Although some clusters show relatively higher coefficients of variation, the overall level remains low, which indicates good consistency of echo wave speed within each cluster.

### Comparison with the conventional method

1. Water hammer period method

To verify the reliability of the wave velocity calibration results, a comparison was carried out under the same well section and the same field conditions. The method proposed in this study inverts the wave velocity by extracting the propagation time delay from the echoes of transient pressure waves generated by perforation excitation, whereas the water hammer period method estimates the wave velocity from the dominant period of the decaying oscillation of the liquid column under water hammer conditions. The two methods involve different excitation mechanisms and signal forms, and therefore they use different characteristic quantities and formula expressions. However, both methods share the same well section geometric scale $L$ and the same in situ medium and boundary conditions, which allows their velocity estimates to be compared in terms of consistency and stability within the same well section.

**Fig. 28:**
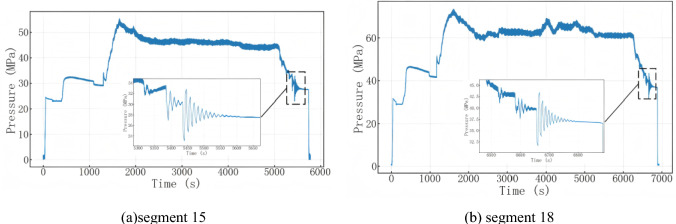
Pressure treatment curve.

**Fig. 29:**
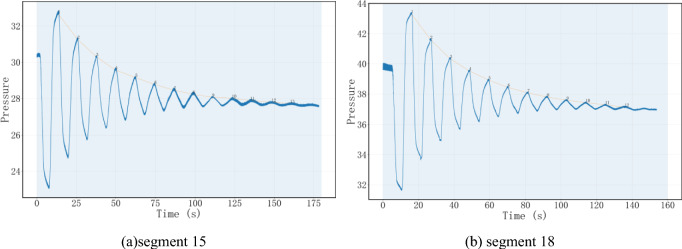
Water hammer period determined by the point marking method.

For the water hammer pressure data, a time window corresponding to the quasi-periodic oscillation after the impact was first selected (Fig. 28). The sequence of peak times was then picked using the point marking method^18^, as shown in Fig. 29. Based on the water hammer period relation $T = \frac{4L}{T}$ reported in the literature, the estimated wave velocities and their statistical indicators are presented in Tables 10 and 11.

**Table 10:** Wave velocity calculated from the water hammer method for Segment 15.

ID	Start_time(s)	Peak_time(s)	Interval(s)	Velocity(m/s)
1	–	13.355	–	–
2	13.355	25.63	12.275	1453.035
3	25.63	37.406	11.776	1514.606
4	37.406	50.081	12.675	1407.179
5	50.081	61.857	11.776	1514.606
6	61.857	74.332	12.475	1429.739
7	74.332	86.307	11.975	1489.436
8	86.307	99.082	12.775	1396.164
9	99.082	110.658	11.576	1540.774
10	110.658	123.632	12.974	1374.749
11	123.632	135.308	11.676	1527.578
12	135.308	147.883	12.575	1418.370
13	147.883	161.056	13.173	1353.982

**Table 11:** Wave velocity calculated from the water hammer method for Segment 18.

ID	Start_time(s)	Peak_time(s)	Interval(s)	Velocity(m/s)
1	–	15.774	–	–
2	15.774	27.040	11.266	1506.835
3	27.040	37.331	10.291	1649.597
4	37.331	48.863	11.532	1472.078
5	48.863	59.242	10.379	1635.610
6	59.242	70.419	11.177	1518.833
7	70.419	80.976	10.557	1608.033
8	80.976	92.242	11.266	1506.835
9	92.242	102.266	10.024	1693.536
10	102.266	114.153	11.887	1428.115
11	114.153	124.621	10.468	1621.704
12	124.621	136.331	11.71	1449.701

Table 12 summarizes the statistical results of wave velocity estimated from perforation pressure and from water hammer pressure under the same well section conditions for Segment 15 and Segment 18, including the mean, standard deviation, and coefficient of variation. It can be seen that the wave velocities obtained by the two methods are of the same order of magnitude in both segments, but differences exist in their mean values and degrees of dispersion. This difference is related to the different excitation mechanisms and signal characteristics of the two types of data: the perforation-based method relies on the travel time delay of transient echoes, whereas the water hammer method relies on the extraction of the period of decaying quasi-periodic oscillations. The wave velocity estimates obtained by the proposed method show lower dispersion, with CV values of 3.3% and 4.6%, respectively, indicating that this method has good stability and consistency under perforation conditions.

**Table 12:** Comparison of wave velocity estimates from perforation pressure and water hammer pressure.

Method	Mean(m/s)	Std (m/s)	CV(100%)
Segment 15 perforation	1544.212	52.435	3.3%
Segment 15 water-hammer	1451.685	64.075	4.4%
Segment 18 perforation	1545.138	71.429	4.6%
Segment 18 water-hammer	1553.716	90.419	5.8%

(2) Cross-correlation method

In this study, the cross-correlation method was introduced as the second comparison scheme. Table 13 summarizes the wave velocity statistics obtained by the cross-correlation method for Segment 15 and Segment 18, and compares them with those obtained by the proposed method. It can be seen that the mean wave velocities obtained by the cross-correlation method are generally lower, and their dispersion is larger.

**Table 13:** Comparison of wave velocity statistics between the perforation-based method and the cross-correlation method.

Method	Mean(m/s)	Std (m/s)	CV(100%)
Segment 15 perforation	1544.212	52.435	3.3%
Segment 15 Xcorr	1327.322	111.581	8.4%
Segment 18 perforation	1545.138	71.429	4.6%
Segment 18 Xcorr	1235.476	70.581	5.7%

### Analysis of distribution patterns and propagation mechanisms

**Fig. 30:**
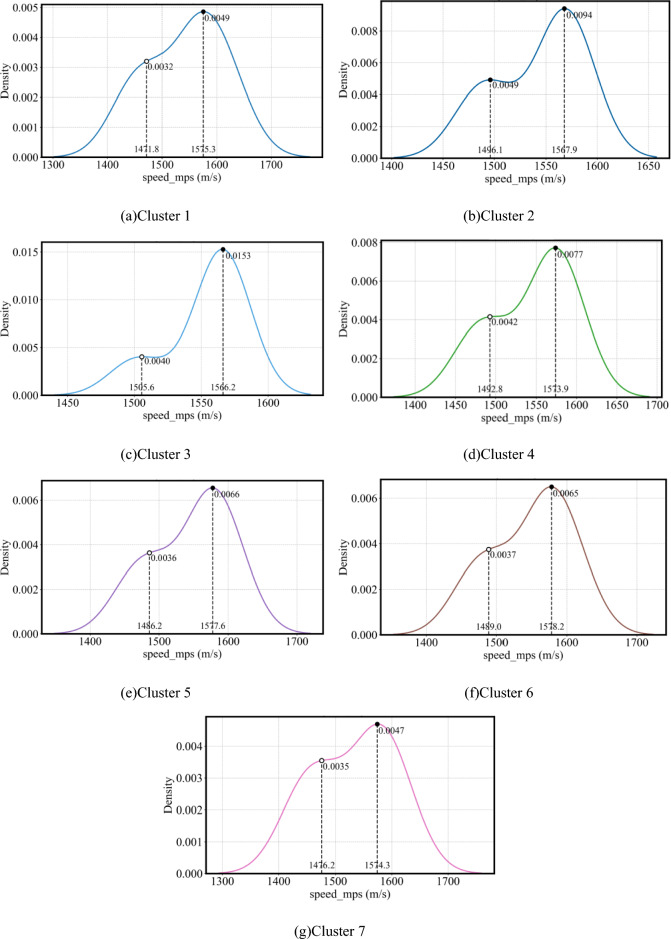
KDE density curves of wave velocity for each cluster in segment 15.

Although the wavefield generated by perforation may contain multiple propagation modes, the observable quantity under the wellhead pressure recording conditions considered in this study mainly reflects the response of wellbore fluid pressure perturbations. Therefore, the multimodality discussed in this paper should be understood as the statistical stratification of equivalent propagation time delays in the wellhead pressure signal, rather than as a strict separation and one-to-one attribution of guided waves, casing waves, or formation-coupled modes. On this basis, kernel density estimation is used to characterize the distribution of equivalent wave velocities, and its possible relationship with different reflection paths or coupling processes is discussed. Figure 30 presents the kernel density distributions of wave speed samples for each perforation cluster in segment 15. The figure shows that the wave speed distributions of different perforation clusters do not strictly follow a single peak pattern, but instead exhibit double peaks or clear shoulder peaks in several clusters. One group of wave speed peaks is concentrated in a higher wave speed range, while another group of peaks is distributed in a relatively lower wave speed range.

Further comparison among perforation clusters shows that although the sample size and distribution shape differ among clusters, the high and low wave speed features can be repeatedly identified in multiple clusters, which indicates that the double peak structure is not caused by abnormal samples from a single cluster, but reflects a certain level of statistical stability.

**Fig. 31:**
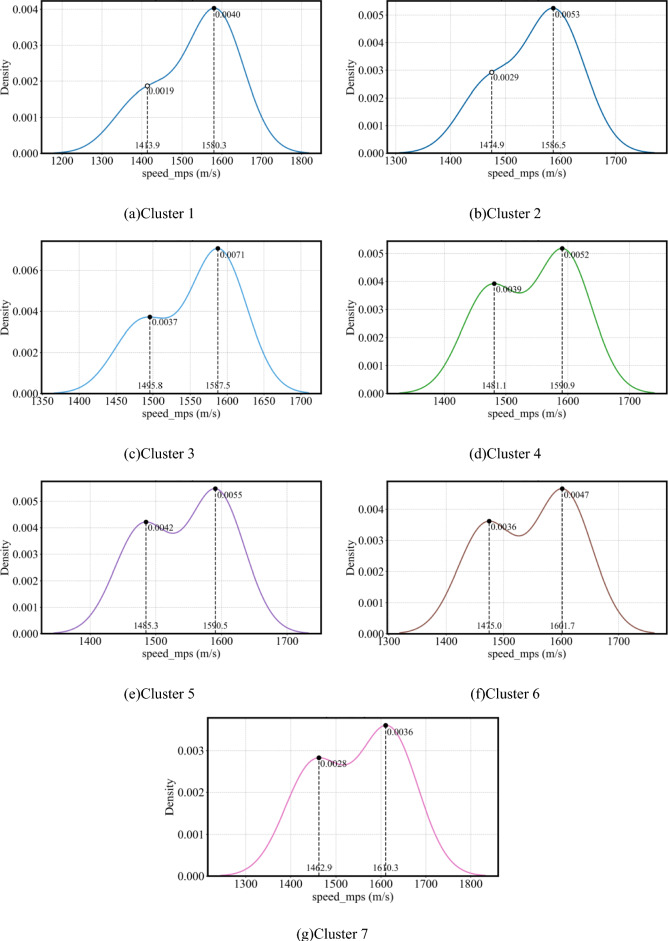
KDE density curves of wave velocity for each perforation cluster in segment 18.

Similar distribution characteristics are also observed in the KDE results of each perforation cluster in segment 18, as shown in Fig. 31. Compared with segment 15, most cluster KDE curves in segment 18 show a clearer bimodal shape, in which the secondary peak has a higher amplitude and a more distinct separation from the primary peak, and in some clusters the two peaks are even relatively close to each other. The consistency in peak locations and distribution patterns between the two segments indicates that this multimodal wave velocity feature has a certain level of repeatability across different segments.

To describe the multi modal features in the wave speed distribution of each perforation cluster in a quantitative manner, the locations of the main peak and the secondary peak in the KDE of each cluster were extracted and summarized, as shown in Table 14. The results show that the main peak identified in different perforation clusters is concentrated in a higher wave speed range, whereas the secondary peak is consistently located in another lower wave speed range, and the two types of peak values do not show clear numerical overlap. From the perspective of variation among clusters, the variation range of the same type of peak across different perforation clusters is relatively limited, and the wave speed difference between the main peak and the secondary peak remains within a similar range for all clusters, which further indicates that these two wave speed features do not correspond to random noise but to two propagation processes with stable differences in scale.

**Table 14:** Statistical results of the KDE main peak and secondary peak positions for each perforation cluster.

Segment ID	cluster_id	peak1_x(m/s)	peak1_y	peak2_x(m/s)	peak2_y
15	1	1575.2529	0.0049	1471.7804	0.0032
	2	1567.8834	0.0094	1496.0698	0.0049
	3	1566.2485	0.0153	1505.6277	0.0040
	4	1573.9384	0.0077	1492.8347	0.0042
	5	1577.6204	0.0066	1486.1537	0.0036
	6	1578.2317	0.0065	1488.9936	0.0037
	7	1574.3148	0.0047	1476.2340	0.0035
18	1	1580.2698	0.0040	1413.9394	0.0019
	2	1586.4824	0.0053	1474.9420	0.0029
	3	1587.4688	0.0071	1492.3273	0.0037
	4	1590.8788	0.0052	1481.0696	0.0039
	5	1590.4604	0.0055	1485.3064	0.0042
	6	1601.7320	0.0047	1474.9654	0.0036
	7	1610.2971	0.0036	1465.8594	0.0028

**Fig. 32:**
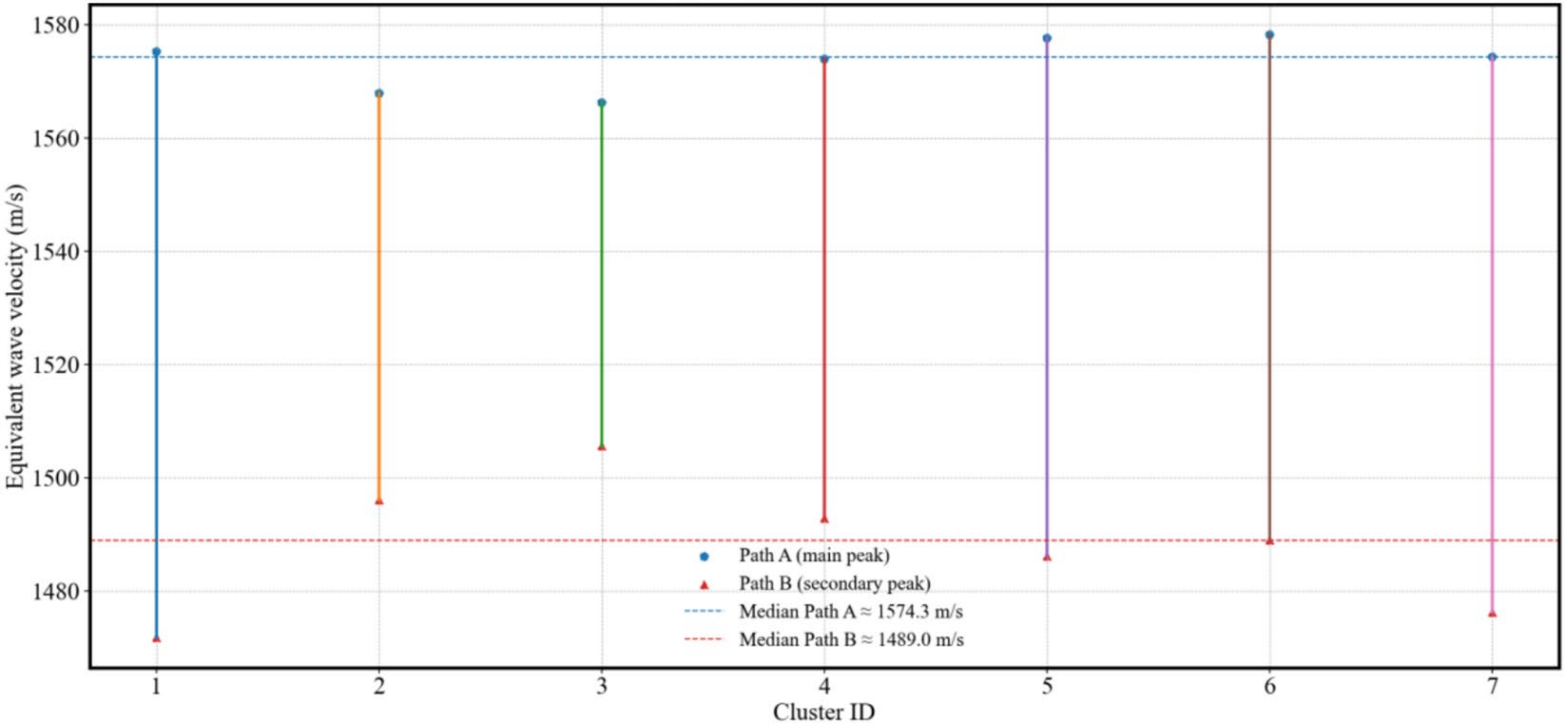
Two stable wave propagation paths identified in different perforation clusters in segment 15.

**Fig. 33:**
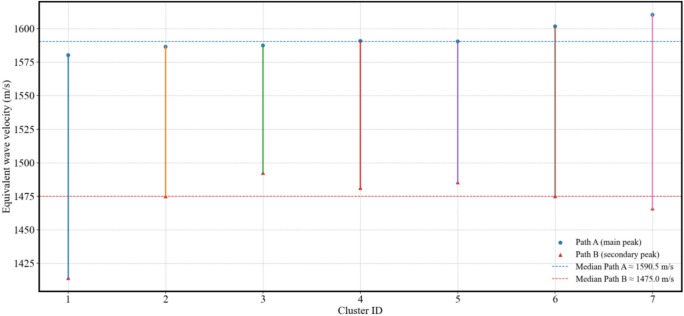
Two stable wave propagation paths identified in different perforation clusters in segment 18.

Based on the statistical results of the main peak and secondary peak positions from the KDE of each perforation cluster, the two types of wave velocity features identified in different perforation clusters are compared across clusters, as shown in Figs. 32 and 33. It can be observed that two types of equivalent wave velocity positions can be identified simultaneously in different perforation clusters, and they form a clear two layer distribution structure across clusters.

It should be noted that although a unified plug distance is used as the geometric reference scale in the wave velocity calculation, the differences in equivalent wave velocity originate from differences in echo arrival time characteristics. The actual propagation process of the perforation impact wave in the wellbore may involve different reflection interfaces and propagation modes, which leads to systematic differences in echo arrival times. After normalization using a fixed distance, these arrival time differences statistically manifest as two stable ranges of equivalent wave velocity.

Considering the wellbore structural features, the high velocity feature is more likely associated with a dominant propagation path formed by strong reflecting interfaces such as bridge plugs, whereas the low velocity feature is more likely related to secondary reflection processes near the perforation location or to coupled propagation between the wellbore and fractures, which shows weaker reflection strength and lower stability.

**Fig. 34:**
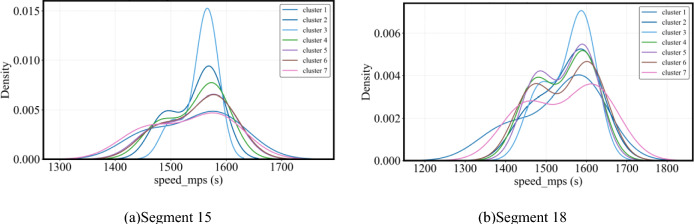
Kernel density estimation curves of wave velocity for perforation clusters.

To verify the velocity distribution features from an overall statistical perspective, velocity samples from all perforation clusters are combined, and kernel density estimation analysis is applied to the merged samples, as shown in Fig. 34. The results indicate that the overall velocity distribution shows a clear two peak structure rather than a single peak pattern. The locations of these two peaks are consistent with the primary peak and secondary peak ranges identified in single cluster and cross cluster analyses, which indicates that the two types of equivalent velocity features show strong consistency at the overall statistical level and further confirms the existence of at least two stable wave propagation paths within the wellbore at the full sample scale.

**Fig. 35:**
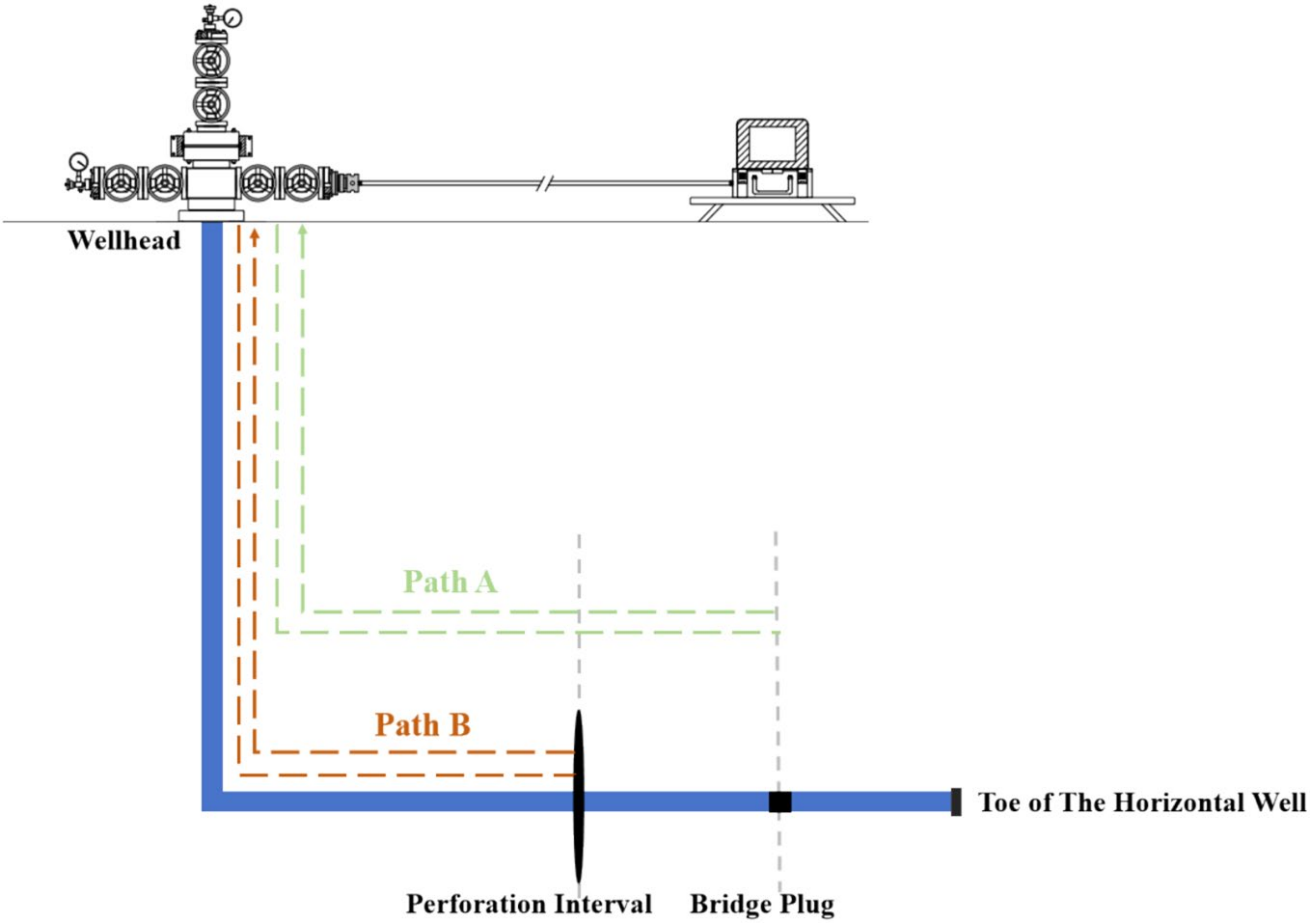
Schematic diagram of multi path propagation of perforation pressure waves.

To explain the bimodal feature observed in the KDE distribution of equivalent wave speed, Fig. 35 presents a schematic diagram of possible multi path propagation of perforation shock waves inside the wellbore. In the schematic diagram, Path A represents the main propagation path dominated by strong reflection interfaces such as the bridge plug, while Path B represents a propagation path related to secondary reflection near the perforation location or to coupling between the wellbore and fractures. Because the echo arrival times differ for the two paths, normalization with a fixed bridge plug distance leads to different ranges of equivalent wave speed.

This study uses the high-frequency transient pressure response recorded by a wellhead pressure sensor, which is more sensitive to perturbations in wellbore fluid pressure. Therefore, the wave velocity calibrated in this paper is defined as the effective pressure-wave speed under wellhead pressure recording conditions, which mainly reflects propagation characteristics dominated by fluid pressure waves. We do not perform strict modal separation of the complex wavefield, although weak modal mixing and weak dispersion may still affect the local waveform.

## Conclusions

This study addresses the difficulty of wave speed calibration in transient wellbore pressure signals during perforation operations. An automatic calibration method for wellbore pressure wave speed based on perforation impact is proposed. The accuracy and stability of the method are systematically verified using both theoretical models and field data. The main conclusions are summarized as follows:

1. A new automatic calibration method for perforation impact wave speed is established. A multi stage preprocessing scheme that includes detrending, Savitzky-Golay smoothing, and Butterworth filtering significantly improves baseline stability and signal to noise ratio. An adaptive peak and trough detection and wave separation strategy enables high accuracy automatic identification of the main impact wave and multiple reflected waves, which overcomes the subjectivity and instability of manual picking.
2. A visual system for wave speed calculation is developed with good user interaction and field applicability. The system is built using the Python language and the PyQt graphical interface framework. It provides graphical and interactive execution of the wave speed calibration process, which effectively reduces subjective judgment and operational complexity, and offers a reliable tool for rapid field analysis of wellbore pressure wave speed.
3. The method demonstrates high accuracy and strong robustness in theoretical models. In the convolution model, the error between the extracted echo intervals and the preset values remains at the millisecond level. In the water hammer model, the identified multiple reflection periods are highly consistent with the theoretical round trip travel time, with relative errors below 0.2 percent, which confirms the time extraction capability and wave speed calibration accuracy of the algorithm.
4. Field perforation data verification shows that the method exhibits good consistency and stability across different perforation stages and perforation cluster scales. For the data from Stage 15 and Stage 18, the identified reflected wave structures are clear and the time intervals are stable. The wave speeds of all clusters are mainly distributed between 1530 and 1555 m/s. The coefficient of variation within clusters is generally below 5 percent, and the average wave speed difference between clusters within a stage is less than plus or minus 15 m/s. In addition, the wave speed statistical distribution at the cluster scale shows stable multimodal features, which indicates that perforation impact waves may propagate along multiple paths in the wellbore and reflects the complexity of the wellbore pressure wave propagation process.
5. The proposed method provides a foundation for fracturing diagnosis and post fracturing evaluation. The automatically calibrated wave speed results can be further used for coupled modeling of perforation, wellbore, and formation systems, as well as for inversion of fracture initiation depth, which supports intelligent fracturing monitoring and real time acoustic diagnosis.

## Data Availability

The data that support the findings of this study are available from the corresponding author upon reasonable request.

## References

[1] Deng, Q., Zhang, H., Li, J., Hou, X. J. & Hao, W. Study of downhole shock loads for ultra-deep well perforation and optimization measures. *Energies* **12**(14), 2743 (2019).

[2] Deng, Q., Zhang, H., Li, J., Hou, X. J. & Zhao, B. X. Numerical investigation of downhole perforation pressure for a deepwater well. *Energies* **12**(19), 3795 (2019).

[3] Chen, C. F. et al. Understanding perforation detonation failure mechanisms based on physicochemical detection and simulation modeling. *Processes* **12**(9), 1971 (2024).

[4] Liu, Y. *Dynamic Response of Perforating Tubing under Impact in HighTemperature and High Pressure Gas Well*. (China University of Petroleum, 2024).

[5] Alexandrov, D., Kashtan, B., Bakulin, A. & Ziatdinov, S. Reflection and transmission of tube waves in cased boreholes with layers and perforations. In *SEG Technical Program Expanded Abstracts 2007*. 3120–3124 (Society of Exploration Geophysicists, 2007).

[6] Li, J. C. et al. Leakage simulation and acoustic characteristics based on acoustic logging by ultrasonic detection. *Adv. Geo Energy Res.* **6**(3), 181–191 (2022).

[7] Wilt, M. et al. Wellbore integrity assessment with casing-based advanced sensing. Proceedings, 43rd Workshop on Geothermal Reservoir Engineering Stanford University, Stanford, California, February 12–14, 2018 SGP-TR-213.

[8] Liang, X. et al. Post-fracturing evaluation and optimization of shut-in of deep shale gas based on high-frequency pressure monitoring: Taking Well DA1 as an example. *Drill. Prod. Technol.* **48**(4), 156–165 (2025).

[9] Deng, Q., Zhang, H., Li, J., Hou, X. J. & Wang, H. Study of downhole shock loads for ultra-deep well perforation and optimization measures. *Energies* **12**(14), 2743 (2019).

[10] Zhang, K. et al. Research on a model for predicting perforating shock loads by numerical simulation in oil and gas wells. *Processes* **13**(8), 2556 (2025).

[11] Li, H. T. et al. Investigation of pressure wave propagation and attenuation characteristics in wellbore gas-liquid two-phase flow. *J. Nat. Gas Sci. Eng.* **35**, 1088–1100 (2016).

[12] Lin, Y. H., Kong, X. W., Qiu, Y. J. & Yuan, Q. J. Calculation analysis of pressure wave velocity in gas and drilling mud two-phase fluid in annulus during drilling operations. *Math. Probl. Eng.* **2013**, 1–17 (2013).

[13] Li, Y. P., Wang, H., Fehler, M. & Fu, Y. Q. Wavefield characterization of perforation shot signals in a shale gas reservoir. *Phys. Earth Planet. Inter.* **267**, 31–40 (2017).

[14] Ye, Q., Sun, H. F., Jin, Z. Q. & Wang, B. Study on shear velocity profile inversion using an improved high frequency constrained algorithm. *Energies* **16**(1), 59 (2022).

[15] Zhang, H. et al. Damage analysis of cement sheath and rock subjected to electrohydraulic shock waves under the perforation completion. *Energy Sci. Eng.* **12**(8), 3289–3307 (2024).

[16] Liu, X. B. et al. A new investigation on optimization of perforation key parameters based on physical experiment and numerical simulation. *Energy Rep.* **8**, 13997–14008 (2022).

[17] Zhang, S. W. & Gao, L. X. Experimental study on pressure wave velocity characteristics in liquid with trace amount of gas pipeline. *Pipeline Tech. Equip.* (1), 1–3, 11 (2016).

[18] Hu, X. D. et al. Water hammer waveform-based experimental study on wave velocity inversion method. *Water Resour. Hydropower Eng.* **53**(5), 106–118 (2022).

[19] Wang, X., Wang, H., Fu, Y., Liu, H. & Chen, P. A novel bypass downlink system for casing sliding sleeve and its laboratory verification. *J. Pet. Sci. Eng.* **201**, 108343 (2021).

[20] Wang, X. et al. A pressure wave recognition and prediction method for intelligent sliding sleeve downlink communication systems based on LSTM. *Energies* **18**(16), 4384 (2025).

[21] Lellouch, A. et al. DAS observations and modeling of perforation-induced guided waves in a shale reservoir. *Lead. Edge* **38**(11), 858–864 (2019).

[22] Li, P. Y. & Jin, G. Using distributed acoustic sensing to characterize unconventional reservoirs via perforation-shot triggered P waves. *Geophysics* **89**(2), P11–P19 (2024).

[23] Bader, M., Clapp, R. G., Nihei, K. T. & Biondi, B. Moment tensor inversion of perforation shots using distributed-acoustic sensing. *Geophysics* **88**(6), WC37–WC45 (2023).

[24] Bader, M., Clapp, R. G., Nihei, K. T. & Biondi, B. Reservoir properties estimation before and after hydraulic stimulation using extended elastic full-waveform inversion and distributed acoustic sensing. *Geophysics* **90**(5), B221–B235 (2025).

[25] Zhang, Z. S., Du, J. & Mavko, G. M. Reservoir characterization using perforation shots: Anisotropy, attenuation and uncertainty analysis. *Geophys. J. Int.* **216**(1), 470–485 (2019).

[26] Yang, Y. D., Huang, F. F. & Kang, S. F. Mechanism of penetration rate improvement in hot dry rock under the coupling of impact load and confining pressure release. *Reserv. Sci.* **2**(1), 52–64 (2026).

[27] Tahir, M. U. & Guo, S. L. Preliminary investigation of fracture behavior during carbon dioxide fracturing of natural hydrogen reservoir with hard-core imperfections. *Reserv. Sci.* **2**(1), 34–51 (2026).

[28] Li, W. S. & Liao, J. Y. Microscopic analysis of flow resistance of oil displacement fluid in reservoir fractures. *Reserv. Sci.* **2**(1), 16–33 (2026).

[29] Shah, S., Hazell, P.J. & Wang, H. et al. Shock wave mitigation in heterogeneous systems: A review. *J. Dynamic Behavior Mater.* **11**, 338–368 (2025).

[30] Gui, M. W. The basics of noise detection and filtering for borehole drilling data. *Open Civ. Eng. J.* **2**(1), 113–120 (2008).

[31] Moosi, S. R., Qajar, J. & Riazi, M. A comparison of methods for denoising of well test pressure data. *J. Pet. Explo. Prod. Technol.* **8**(4), 1519–1534 (2018).

